# Photophysical Properties of Free, Zinc‑, and Pyridine- Chlorin e6 Trimethyl Ester: A Computational Study with Vibrational Effects

**DOI:** 10.1021/acsomega.5c10268

**Published:** 2026-03-24

**Authors:** Marlon D. Suárez Ruiz, Martha C. Daza, Markus Doerr

**Affiliations:** Grupo de Bioquímica Teórica, 28014 Universidad Industrial de Santander, Bucaramanga 680002, Colombia

## Abstract

Chlorophyll derivatives, such as chlorin e6, are natural products with promising properties as photosensitizers (PS); they are highly abundant, and their extraction is a relatively straightforward process. Therefore, they serve as an ideal starting point for the rational design of new photosensitizers with enhanced properties. In this project, the photophysics of chlorin e6 trimethyl ester (TMEe6), along with its derivatives with zinc (ZnTMEe6) and pyridine (PyrTMEe6), have been computationally characterized. Spectra and transition rate constants for absorption, fluorescence, phosphorescence, and intersystem crossing were computed using the implementation of the analytical solution of Fermi’s golden rule via the path integral formalism including vibrational effects. The Adiabatic Hessian model was employed in conjunction with TDDFT CAM-B3LYP/def2-SVP geometries, Hessians, and energies of the involved singlet and triplet states. The Tamm–Dancoff approximation (TDA) was used to optimize the triplet states due to triplet instabilities. The computed spectra are reasonably close to the experimental ones, with some differences in vibrational bands, but good agreement with (0–0) bands, with deviations of less than 0.05 eV in band maxima, and up to 0.2 eV if TDA/TDDFT is used. Calculated rates for fluorescence and ISC are of the same order of magnitude as the experimental data available, which is approximately 10^8^ s^–1^. Our results suggest that the main intersystem crossing (ISC) channel for TMEe6 and PyrTMEe6 involves coupling between S_1_ ⇝ T_2_, and for ZnTMEe6 between S_1_ ⇝ T_3_. Particularly, the addition of zinc leads to a destabilization of the T_3_ state, making the S_1_ and T_3_ states almost isoenergetic. This change results in an enhancement of the ISC rate by a factor of 2.19 when compared to TMEe6. Pyridine addition enhances ISC rates for channels S_1_ ⇝ T_1_ and S_1_ ⇝ T_3_, but not for the main channel S_1_ ⇝ T_2_. This ultimately increases the total ISC rate by 41%. This methodology provides a better understanding of the photophysics of these molecules that could not be observed with the usual energy gaps and spin–orbital coupling matrix elements. It could therefore aid in the development of a rationally designed synthetic protocol for porphyrinoid photosensitizers.

## Graphical abstract

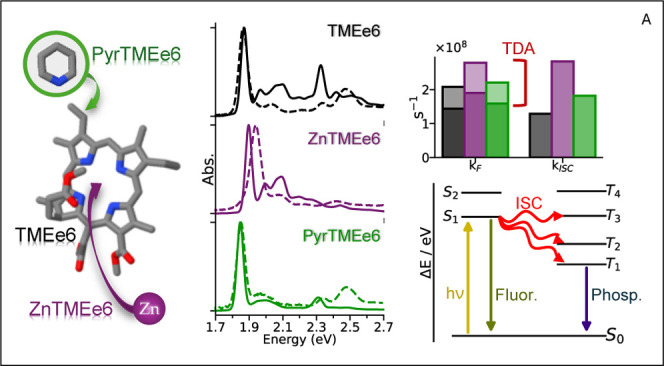

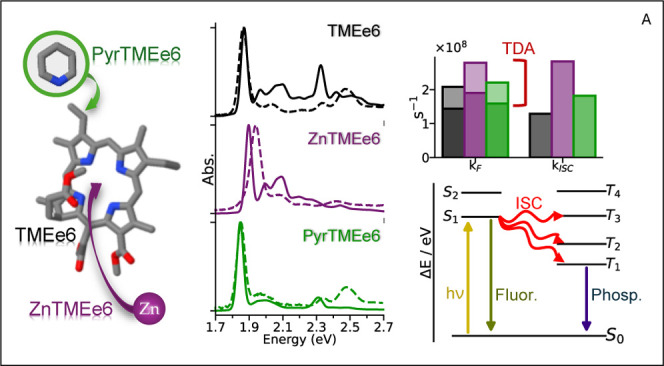

## 1 Introduction

Chlorophylls and other porphyrinic systems are referred to as “pigments of life”. These molecules have been extensively studied over the last century. Currently, there are entire journals and series of books dedicated to them such as the Journal of Porphyrins and Phthalocyanines (JPP), The Porphyrins,^1^ and The Porphyrin Handbook.^2^ Additionally, numerous reviews compile different chlorophyll derivatives and approaches upon study.^3–5^ Several derivatives have been studied as photosensitizers (PS) of natural origin,^4^ with their properties being fine-tuned depending on the field of application. Examples of such fields include photodynamic therapy (PDT), photothermal therapy (PTT), antiviral and antimicrobial treatments, wastewater treatment, and bioimaging. In this context, their properties make them particularly attractive for use in PDT cancer therapy: these molecules exhibit absorption in the treatment window (600–800 nm), low dark cytotoxicity, and a high quantum yield of singlet oxygen, around 0.65.^6^ Research on chlorin e6, a common chlorophyll derivative, has focused on fine-tuning its properties.^7,8^ One example of this is Talaporfin,^9^ a commercial photosensitizer (PS) derived from it that is employed in anticancer research. Usual trends in these modifications are the extension of conjugated π system in order to reduce the energy gap between HOMO and LUMO orbitals, which results in a bathochromic shift of the absorption Q bands;^10–15^ and the formation of metal complexes with heavy atoms, which is known to enhance intersystem crossing (ISC).^14,16–20^

Nevertheless, the performance of a photosensitizer is dependent on its photophysical properties, which are experimentally measured. However, their computational characterization allows synthesis procedures to be bypassed, providing insights into their properties. Typically, computational studies use time-dependent density functional theory (TDDFT) to compute electronic structure and spin–orbital coupling matrix elements (SOCMEs), placing particular emphasis on the energy gaps between the involved states and the magnitude of SOCMEs that are then compared with those of other known photosensitizers.^16,21–23^ This methodology has a low computational cost, enabling the study of a series of derivatives. However, this method does not explain the performance of organic photosensitizers, such as tetrapyrroles. Tetrapyrroles exhibit high quantum yields of singlet oxygen, exceeding 0.6, yet possess low SOCMEs, below 1 cm^–1^.^7,24^ Therefore, this simple approach alone is insufficient. To gain a deeper understanding of these properties, it is necessary to compute transition rates and spectra that incorporate additional effects, such as Herzberg–Teller effects and Duschinsky rotations.^25,26^ Fortunately, novel methods have been developed in the last 15 years that enable the computation of those properties in large molecules, such as chlorophylls.^27–31^

In this paper, we present a computational study of the photophysical properties of chlorin e6 trimethyl ester (TMEe6), its zinc­(II) complex derivative (ZnTMEe6), and its piridinic derivative (PyrTMEe6). Their structure is shown in Figure 1. Experimental research has recently been conducted on the semisynthesis of these molecules.^32–34^ This work aims to provide new insights into the photophysical properties of these molecules by using state-of-the-art computational methods that incorporate vibrational and thermal effects.^27–30^ These will then be compared with the available experimental data. Recently, we outlined a straightforward computational protocol based on DFT and TD-DFT calculations.^35^ As demonstrated below, calculated spectra and rates are reasonably close to the experimental data. These results highlight the impact of Herzberg–Teller (HT) effects on calculated rates. This work reproduces the effect of adding zinc and pyridine to calculated spectra, making it a viable tool for the computer-aided design of promising porphyrinoid photosensitizers.

**Figure 1:**
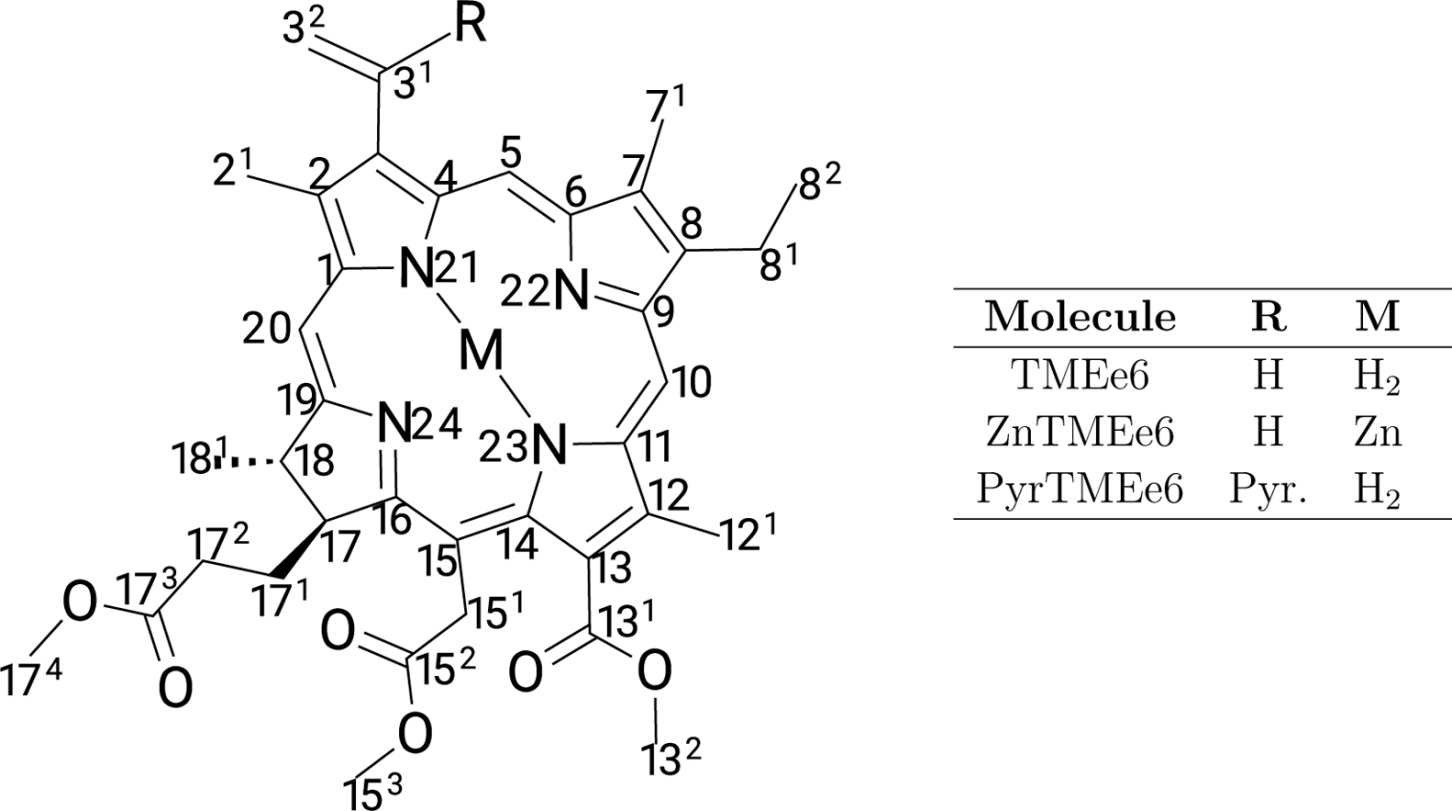
Chemical structure of chlorin e6 trimethyl ester (TMEe6) and their derivatives with zinc (ZnTMEe6) and pyridine (PyrTMEe6).

## 2 Computational Details

As the first step, a conformational exploration was conducted to identify the minimum energy conformer using CREST^36^ and CENSO.^37^ CREST (Conformer–Rotamer Ensemble Sampling Tool) and CENSO (Conformer–Rotamer Ensemble Sorting) provide a robust workflow for conformational analysis. CREST employs RMSD-biased metadynamics with GFN-based semiempirical and force-field methods to efficiently generate thermodynamically relevant ensembles of conformers and rotamers. These ensembles are then refined by CENSO, which reranks the structures at higher levels of theory, typically DFT with solvation models, to yield reliable energetic hierarchies and Boltzmann populations. Together, they enable accurate free energy descriptions and improve the prediction of thermodynamic and spectroscopic properties.

The minimum energy conformer was then selected as the starting structure for the optimization for the ground (S_0_) states in ORCA 5.0.4 ^38^ using density functional theory (DFT). The functional CAM-B3LYP^39^ and the Ahlrichs basis set def2-SVP^40^ were selected for all optimizations. Using the same functional and basis set, the first singlet (S_1_, S_2_, S_3_) and triplet (T_1_, T_2_, T_3_, T_4_) excited states were optimized starting from the optimized ground state, employing time-dependent DFT (TDDFT).^41^ Triplet geometries were optimized using the Tamm–Dancoff approximation (TDA)^41^ to avoid triplet instabilities.^42^ However, the optimizations of T_2_ (TMEe6 and PyrTMEe6) and T_3_ (ZnTMEe6) were carried out using full-TDDFT, as TDA failed to converge due to root-flipping involving higher excited states. Single-point TDDFT calculations using CAM-B3LYP/def2-SVP were performed to compute energies, oscillator strengths, and transition dipole moments using both full-TDDFT and TDA/TDDFT with 5 roots for each optimized structure. The full-TDDFT and TDA/TDDFT energies were used in the computation of absorption and fluorescence spectra, while phosphorescense and ISC used the TDA/TDDFT energies. Absorption was calculated for the S_0_ → S_1_, S_2_ transitions, fluorescence for the S_1_ → S_0_ transition, and phosphorescence for the T_1_ → S_0_ transition as the average over their three spin sublevels (−1,0,1). The chain of spheres approach (COSX)^43^ and the resolution of identity approximation (RIJ)^44^ were used to speed up calculations with its auxiliary basis set def2/J.^45^ The conductor-like polarizable continuum solvation model (CPCM)^46^ was employed for all calculations using CH_2_Cl_2_ as solvent along with the D3-BJ dispersion correction proposed by Grimme’s group.^47,48^ Vibrational frequencies were used to characterize the nature of the optimized geometries of the ground and excited states as minima using the same level of theory, CAM-B3LYP/def2-SVP, and to calculate zero-point energy (ZPE) corrections.

Transition rates and spectra with vibrational resolution were calculated using the excited states dynamics (ESD) module implemented in ORCA 5.0.4.^49^ Here we briefly describe it, and we refer to the original works for a complete explanation.^28,29^ This module solves analytically Fermi’s golden rule (eq 1) using a path integral approach, where the total rate constant is the sum over all possible transitions between states that are thermally accessible

1 $$k_{\text{if}}=\alpha |\left\langle \Psi_{i}\left|Ô\right|\Psi_{f}\right\rangle {|}^{2}\delta \left(\omega_{\text{if}}\right),k_{obs}=\sum_{\text{if}}k_{\text{if}}\left(e^{-\varepsilon_{i}/k_{B}T}\right)/Z$$

Here α is a prefactor represented as 4ω^3^ *n* ^2^/3*ℏc* ^3^, for radiative processes, where *n* is the refractive index of the medium, or as 2π/*ℏ* for intersystem crossing (ISC). The operator Ô in eq 1 depends on the process involved in the transition: the transition dipole moment operator μ̂, in the case of radiative processes such as absorption, fluorescence, or the spin–orbit coupling operator ${Ĥ}_{\text{SO}}$ , where a change in spin is involved, as in the case of intersystem crossing. Particularly, phosphorescence includes both the *Ĥ*_SO_ and μ̂ operators, because first occurs a mix of pure spin states, which are then used to predict the emission of a photon.^28,50^ The ω_if_ inside Dirac’s delta is the energy difference between initial and final states expressed as a Bohr frequency. Depending on the process it is required to include the energy of absorbed or emitted photon as

$$\omega_{\text{if}}=\{\begin{array}{ll} \left(E_{i}-E_{f}\right)/\hbar & \text{for}\;ISC\\ \left(E_{i}-E_{f}+\hbar \nu \right)/\hbar & \text{for}\;absorption\\ \left(E_{i}-E_{f}-\hbar \nu \right)/\hbar & \text{for}\;emission \end{array}$$ 2

The Dirac delta function is expressed in its Fourier transformation form as δ­(ω) = 1/2π∫*e* ^iω*t*^d*t*. Additionally, it is possible to include vibronic coupling using the Taylor series expansion up to first order as is shown for the matrix element of the transition dipole operator.

3 $${\mathrm{\mu ⃗}}^{e}\left(Q\right)={\mathrm{\mu ⃗}}_{0}^{e}+{\sum_{i}\frac{\partial {\mathrm{\mu ⃗}}^{e}}{\partial Q_{i}}|}_{Q=0}Q_{i}$$

The zeroth-order term in eq 3 is known as the Franck–Condon (FC) approximation^51,52^ which is usually enough to calculate allowed transitions. The first-order term is known as the Herzberg–Teller (HT) effect^53^ which explicitly includes the vibrational dependence on the matrix element. It is required to calculate weakly allowed or forbidden transitions.^26,30^ The inclusion of these effects is essential for an accurate description of Q bands in porphyrins and their derivatives.^25,26,30^ Furthermore, this expansion can be equally applied to include the vibrational dependence of the spin–orbit coupling for ISC.^28^ This method is gaining popularity and allows computing a dynamic property, the transition rate constant, based on static properties.^54–56^

Spin–orbit matrix elements (SOCMEs) were calculated using the mean-field approximation for two-electron integrals of the Breit–Pauli spin–orbit Hamiltonian^57^ known as AMFI,^58^ implemented in ORCA.^59^ The Adiabatic Hessian (AH) model, Herzberg–Teller effects and Duschinsky rotations were employed in our calculations. To achieve an adequate band shape, it was required to exclude low-frequency anharmonic modes below 350 cm^–1^, as was done in our previously published work.^35^ These modes present difficulties for both the harmonic approximation and the conventional Duschinsky transformation. However, for the calculation of ISC rate constants, we discarded vibrational modes below 100 cm^–1^. The spectra were calculated using a Gaussian broadening function with an inhomogeneous bandwidth of 150 cm^–1^, while a vibronic progression was calculated using an inhomogeneous bandwidth of 1 cm^–1^. ISC rates were calculated using a Gaussian broadening function with an inhomogeneous bandwidth of 10 cm^–1^. All other settings were set to their default values.

## 3 Results and Discussion

### 3.1 Conformational Exploration

The minimum energy conformers found after refinement with CENSO are shown in Figure 2. These structures show that the chain at C17 (see Figure 1) bends over the macrocycle. In previous theoretical studies this fragment has been replaced with a hydrogen or methyl group since it is not conjugated to the macrocycle ring.^27,60^ Higher energy conformers exhibit methyl, vinyl (C3 in Figure 1), and ethyl (C8 in Figure 1) rotations (see Figure S16 in Supporting Information for further details). The C3 vinyl and C8 ethyl orientations are in agreement with the single-crystal X-ray data of Talaporfin. It should be noted that only one X-ray work has been documented in the literature.^9^

**Figure 2:**
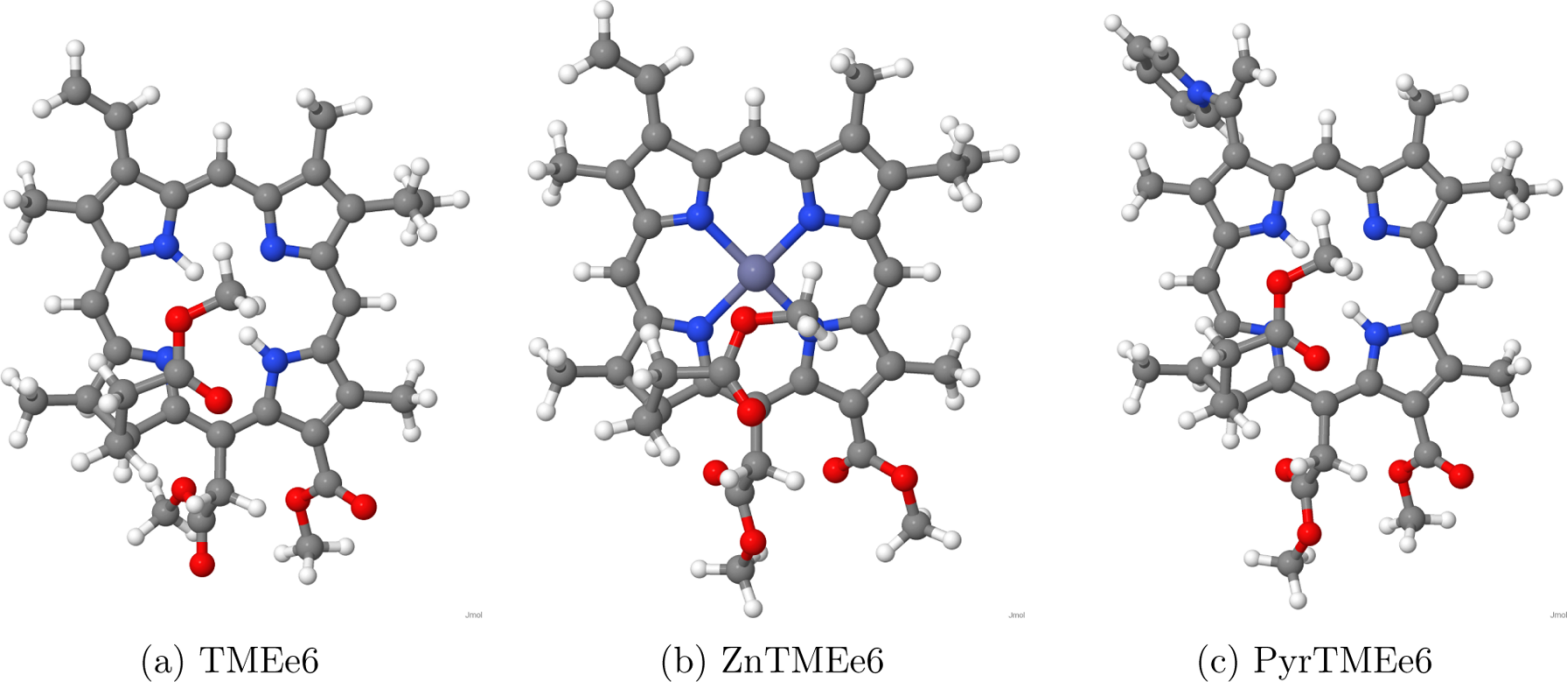
Minimum energy conformers found in the conformational exploration with CREST-CENSO.

### 3.2 Ground and Excited States Optimizations

Cartesian coordinates for all optimized structures are provided in the Supporting Information (Supporting Information). Optimized structures were characterized as minima based on vibrational frequencies, with no imaginary modes detected. Single-point calculations were performed to characterize their electronic structure. The geometries of the excited and ground states differ by less than 5 pm for bond lengths, primarily due to the elongation of the methene bridges.

### 3.3 Excited States Characterization

According to the four-orbital Gouterman model, the absorption bands of porphyrin derivatives arise from transitions between two HOMO orbitals (HOMO and HOMO – 1) and two LUMO orbitals (LUMO and LUMO + 1).^61–63^ The transitions described by this model explain the two characteristic regions of the absorption spectrum, a low-energy region called Q, located between 500 and 700 nm, and a higher-energy region, called B or Soret, located between 350 and 450 nm. Chlorin e6 trimethyl ester follows this model, showing two bands in the Q region, Q_*y*_ at 660 nm and Q_*x*_ at 500–530 nm, and a wide Soret band at 400 nm.^32,64,65^

Tables 1 and 2 illustrates the characteristics of the excited states character at the optimized ground state for each molecule using full-TDDFT and TDA/TDDFT. These results agree with the Gouterman model and previous computational studies.^22,27,66^

**Table 1:** Full-TDDFT/CAM-B3LYP­(CPCM)/def2-SVP Electronic Structures at the Ground State Geometry S_0_ of Chlorin e6 Trimethyl Ester Derivatives in CH_2_Cl_2_ ^a^

	state	VEEs (eV)[*f*_osc_]	λ (nm)	|μ|^2^ (a.u.)^2^	attribution
TMEe6	S_1_(Q_*y*_)	2.067 [0.423]	600	8.35	HOMO → LUMO:0.85
	S_2_(Q_*x*_)	2.490 [0.145]	498	2.37	HOMO – 1 → LUMO:0.70, HOMO → LUMO + 1:0.24
	S_3_(B_*y*_)	3.149 [1.663]	394	21.56	HOMO → LUMO + 1:0.72, HOMO – 1 → LUMO:0.25
	S_4_(B_*x*_)	3.308 [1.510]	375	18.63	HOMO – 1 → LUMO + 1:0.83
	T_1_	0.919	-	-	HOMO → LUMO:0.92
	T_2_	1.432	-	-	HOMO – 1 → LUMO:0.91
	T_3_	2.032	-	-	HOMO → LUMO + 1:0.68, HOMO – 1 → LUMO:0.28
	T_4_	2.472	-	-	HOMO – 1 → LUMO + 1:0.83
ZnTMEe6	S_1_(Q_*y*_)	2.075 [0.519]	598	10.21	HOMO → LUMO:0.91
	S_2_(Q_*x*_)	2.556 [0.139]	485	2.22	HOMO – 1 → LUMO:0.75, HOMO → LUMO + 1:0.20
	S_3_(B_*y*_)	3.174 [1.513]	390	19.45	HOMO → LUMO + 1:0.76, HOMO – 1 → LUMO:0.20
	S_4_(B_*x*_)	3.480 [1.324]	356	15.53	HOMO – 1 → LUMO + 1:0.75, HOMO – 2 → LUMO:0.12
	S_5_ CT(π, π*)	3.548 [0.142]	349	1.63	HOMO – 2 → LUMO:0.67, HOMO – 1 → LUMO + 1:0.12
	S_6_(σ, π*)	3.640 [0.032]	340	0.36	HOMO – 4 → LUMO:0.73, HOMO – 5 → LUMO:0.20
	T_1_	1.062	-	-	HOMO → LUMO:0.80
	T_2_	1.604	-	-	HOMO – 1 → LUMO:0.76, HOMO → LUMO:0.10
	T_3_	2.107	-	-	HOMO → LUMO + 1:0.82
	T_4_	2.511	-	-	HOMO – 2 → LUMO:0.52, HOMO – 2 → LUMO + 1:0.10
PyrTMEe6	S_1_(Q_*y*_)	1.921 [0.439]	645	9.33	HOMO → LUMO:0.88
	S_2_(Q_*x*_)	2.408 [0.095]	515	1.62	HOMO – 1 → LUMO:0.72, HOMO → LUMO + 2:0.25
	S_3_ CT(π, π*)	3.027 [0.333]	410	4.50	HOMO → LUMO + 1:0.75, HOMO → LUMO + 2:0.13
	S_4_(B_*y*_)	3.105 [1.300]	399	17.10	HOMO → LUMO + 2:0.58, HOMO → LUMO + 1:0.19
	S_5_(B_*x*_)	3.258 [1.407]	381	17.63	HOMO → LUMO + 2:0.81
	T_1_	0.831	-	-	HOMO – 1 → LUMO:0.66, HOMO → LUMO + 2:0.17
	T_2_	1.141	-	-	HOMO → LUMO:0.86
	T_3_	2.026	-	-	HOMO → LUMO + 2:0.74, HOMO – 1 → LUMO:0.19
	T_4_	2.446	-	-	HOMO – 1 → LUMO + 2:0.86

a VEE is the vertical excitation energy of each excited state in eV. *f*_osc_ is the oscillator strength. λ is the wavelength in nm associated with each VEE. |μ|^2^ is the square of transition dipole moment calculated for each excited state.

**Table 2:** TDA/TDDFT/CAM-B3LYP­(CPCM)/def2-SVP Electronic Structures at the Ground State Geometry S_0_ of Chlorin e6 Trimethyl Ester Derivatives in CH_2_Cl_2_ ^a^

molecule	state	VEEs (eV)[*f*_osc_]	λ (nm)	|μ|^2^ (a.u.)^2^	attribution
TMEe6	S_1_(Q_*y*_)	2.226 [0.466]	557	8.54	HOMO → LUMO:0.80, HOMO – 1 → LUMO + 1:0.12
	S_2_(Q_*x*_)	2.626 [0.121]	472	1.88	HOMO – 1 → LUMO:0.64, HOMO → LUMO + 1:0.29
	S_3_(B_*y*_)	3.454 [2.429]	359	28.70	HOMO → LUMO + 1:0.66, HOMO – 1 → LUMO:0.28
	S_4_(B_*x*_)	3.516 [2.137]	353	24.80	HOMO – 1 → LUMO + 1:0.76, HOMO → LUMO:0.12
	T_1_	1.538	-	-	HOMO → LUMO:0.70, HOMO – 1 → LUMO:0.20
	T_2_	1.763	-	-	HOMO – 1 → LUMO:0.70, HOMO → LUMO:0.23
	T_3_	2.191	-	-	HOMO → LUMO + 1:0.88
	T_4_	2.612	-	-	HOMO – 1 → LUMO + 1:0.87
ZnTMEe6	S_1_(Q_*y*_)	2.235 [0.592]	555	10.81	HOMO → LUMO:0.87
	S_2_(Q_*x*_)	2.680 [0.114]	463	1.75	HOMO – 1 → LUMO:0.68, HOMO → LUMO + 1:0.27
	S_3_(B_*y*_)	3.443 [1.977]	360	23.44	HOMO → LUMO + 1:0.67, HOMO – 1 → LUMO:0.23
	S_4_ CT(π, π*)	3.610 [0.758]	343	8.57	HOMO – 2 → LUMO:0.60, HOMO – 1 → LUMO + 1:0.18
	S_5_(σ, π*)	3.658 [0.097]	339	1.08	HOMO – 4 → LUMO:0.64, HOMO – 5 → LUMO:0.23
	S_6_(B_*x*_)	3.706 [1.454]	334	16.01	HOMO – 1 → LUMO + 1:0.62, HOMO – 2 → LUMO:0.11
	T_1_	1.486	-	-	HOMO → LUMO:0.90
	T_2_	1.919	-	-	HOMO – 1 → LUMO:0.91
	T_3_	2.295	-	-	HOMO → LUMO + 1:0.90
	T_4_	2.730	-	-	HOMO – 1 → LUMO + 1:0.79
PyrTMEe6	S_1_(Q_*y*_)	2.103 [0.485]	589	9.33	HOMO → LUMO:0.82, HOMO – 1 → LUMO + 2:0.14
	S_2_(Q_*x*_)	2.557 [0.072]	485	1.62	HOMO – 1 → LUMO:0.64, HOMO → LUMO + 2:0.32
	S_3_ CT(π, π*)	3.046 [0.013]	407	0.18	HOMO → LUMO + 1:0.95
	S_4_(B_*y*_)	3.105 [2.284]	364	27.34	HOMO → LUMO + 2:0.59
	S_5_(B_*x*_)	3.258 [1.846]	356	21.63	HOMO – 1 → LUMO + 2:0.63, HOMO → LUMO:0.11
	T_1_	1.448	-	-	HOMO → LUMO:0.95
	T_2_	1.634	-	-	HOMO – 1 → LUMO:0.89
	T_3_	2.149	-	-	HOMO → LUMO + 2:0.90
	T_4_	2.559	-	-	HOMO – 1 → LUMO + 2:0.90

a VEE is the vertical excitation energy of each excited state in eV. *f*_osc_ is the oscillator strength. λ is the wavelength in nm associated with each VEE. |μ|^2^ is the square of transition dipole moment calculated for each excited state.

The Frontier molecular orbitals are shown in Figure 3. These orbitals show some contributions from the vinyl and pyridine substituents, with minimal contributions from the methyl-esters. This explains the little effect that peripheral substituents at C13, C15 and C17 chains have on previously reported photophysical processes of chlorin e6 and chlorin p6.^65,67,68^ There is a negligible contribution of zinc orbitals to the Frontier molecular orbitals, except for the HOMO – 1, which exhibits some delocalization over the metal center.

**Figure 3:**
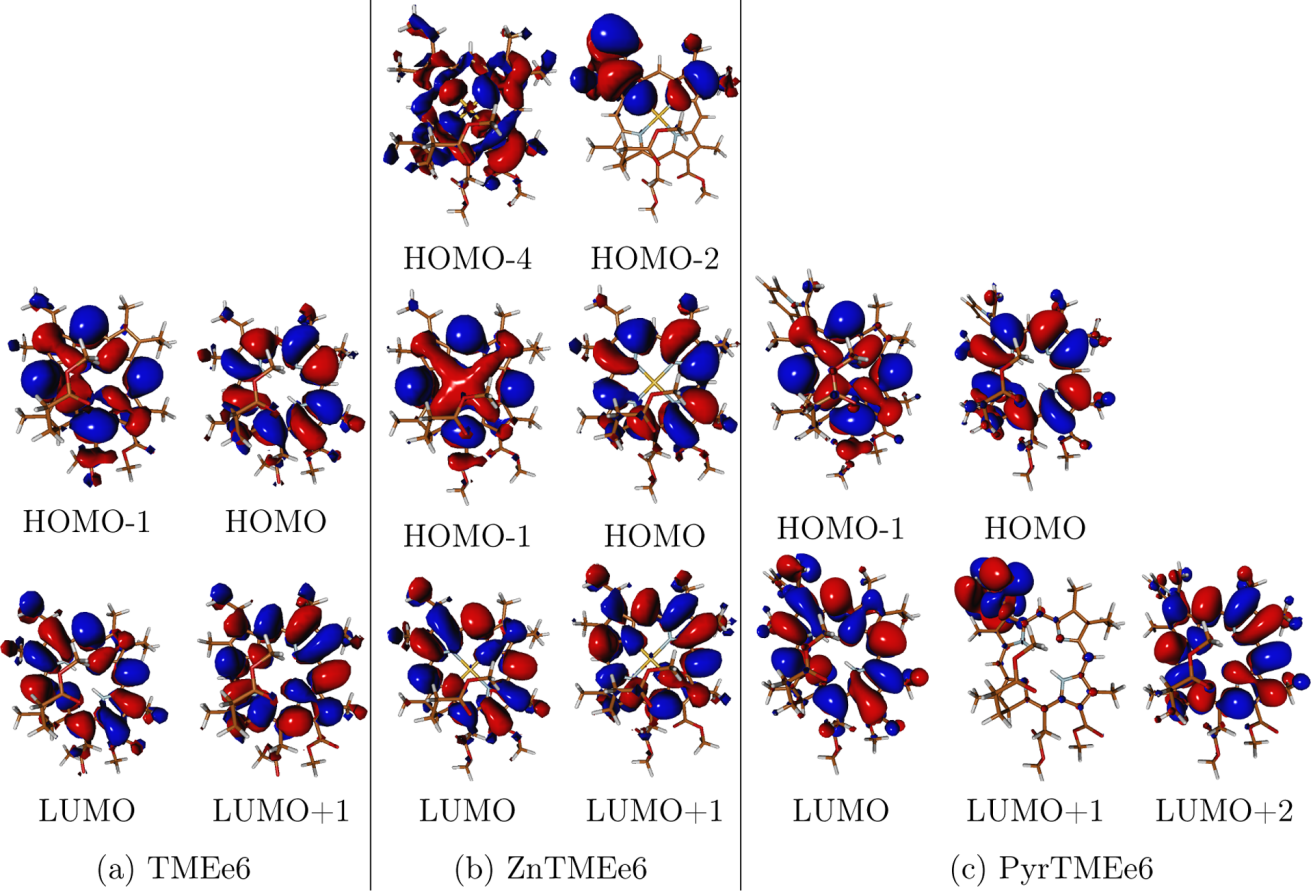
Frontier molecular orbitals at S_0_ geometry of TMEe6, ZnTMEe6, and PyrTMEe6 involved in low-lying transitions. Calculated with TDA/CAM-B3LYP/def2-SVP.

The two non-Gouterman states, S_5_ CT­(π, π*) and S_6_(σ, π*), for ZnTMEe6 in Table 1 appear before than the Gouterman S_6_(π, π*) state when TDA/TDDFT is used, as shown in Table 3. A comparison between full-TDDFT and TDA/TDDFT reveals that when TDA is used, the Gouterman states exhibit a consistent blue shift of approximately 0.2 eV, while the two non-Gouterman states show a more minor blue shift of around 0.05 eV. The triplet states shows a larger shift for T_1_ and T_2_ states, over 0.4 and 0.3 eV for ZnTMEe6, as shown in Table 3. This effect is more pronounced for TMEe6 and PyrTMEe6, with shifts over over 0.6 eV for T_1_ and around 0.3–0.5 eV for T_2_. These energy differences for triplet states when using a hybrid functional such as CAM-B3LYP are well-documented and are attributable to triplet instabilities that cause unphysical low energies for triplet states.^42,69–71^ A similar pattern is observed after comparing TDDFT results with DFT/MRCI results for a free-base porphyrin and separately with DLPNO-STEOM-CCSD results for a chlorophyll derivative.^24,27^

**Table 3:** Ordering of Low-Lying Excited States Using Vertical Excitation Energies for ZnTMEe6 at the Geometry of the Ground State with full-TDDFT and TDA/TDDFT

full-TDDFT				TDA/TDDFT			
state	character^a^	*E* (eV)	*f*_osc_	state	character^a^	*E* (eV)	*f*_osc_
S_1_	(π, π*)	2.075	0.519	S_1_	(π, π*)	2.240	0.592
S_2_	(π, π*)	2.556	0.139	S_2_	(π, π*)	2.680	0.114
S_3_	(π, π*)	3.174	1.513	S_3_	(π, π*)	3.443	1.977
S_4_	(π, π*)	3.480	1.324	S_4_	CT(π, π*)	3.610	0.758
S_5_	CT(π, π*)	3.548	0.142	S_5_	(σ, π*)	3.658	0.097
S_6_	(σ, π*)	3.640	0.032	S_6_	(π, π*)	3.706	1.454
T_1_	(π, π*)	1.062	-	T_1_	(π, π*)	1.448	-
T_2_	(π, π*)	1.604	-	T_2_	(π, π*)	1.919	-
T_3_	(π, π*)	2.107	-	T_3_	(π, π*)	2.295	-
T_4_	(π, π*)	2.511	-	T_4_	(π, π*)	2.730	-

a Character based on molecular orbitals.

PyrTMEe6 derivative shows a new charge transfer (CT) state S_3_ (π, π*) between the chlorin π conjugated macrocycle and the pyridine aromatic ring that lies between the second and third Gouterman state. Usually CT states are of great interest because they are known to promote ISC. The change in orbital angular momentum from a CT state to other nearby excited states of different character enhances ISC, in accordance with El-Sayed’s rules.^53^ Unfortunately, this state is too high in energy to consider it as an effective ISC channel. As it is clear from Table 3, the use of TDA does not affect the character of states involved in ISC; however, it does influence their energies. Given that ISC rates are sensitive to the energy gap between singlets and triplets, and that triplets were optimized using TDA, energies calculated with TDA for singlets were used to compute ISC constant rates in order to maintain consistency. Consequently, absorption and fluorescence were calculated using both Tamm-Dancoff approximation and full-TDDFT energies, and their differences were analyzed.

### 3.4 Calculated Absorption, Fluorescence, and Phosphorescence Spectra

In chlorins the UV–vis absorption spectrum is typically divided into two regions, Q and B. B bands are the most intense and energetic bands located around 400 nm. Q bands are the most relevant for biological applications such as light penetration for treating deep-seated tumors. This property may be enhanced by fine-tuning the molecular structure to achieve a red-shift in the Q bands.^14,72^ Consequently, in the present study, we focus on the calculated spectra for Q bands. We compare the calculated spectra for the Q bands with the experimental data, as shown in Figure 4. The use of TDA results in a consistent blueshift of 0.2 eV for Q-bands that could be related to an overestimation of singlet states when using CAM-B3LYP.^42^ As shown in the Supporting Information in figures S4–S9, this only displaces the calculated spectra, while the vibrational bands remain unchanged.

**Figure 4:**
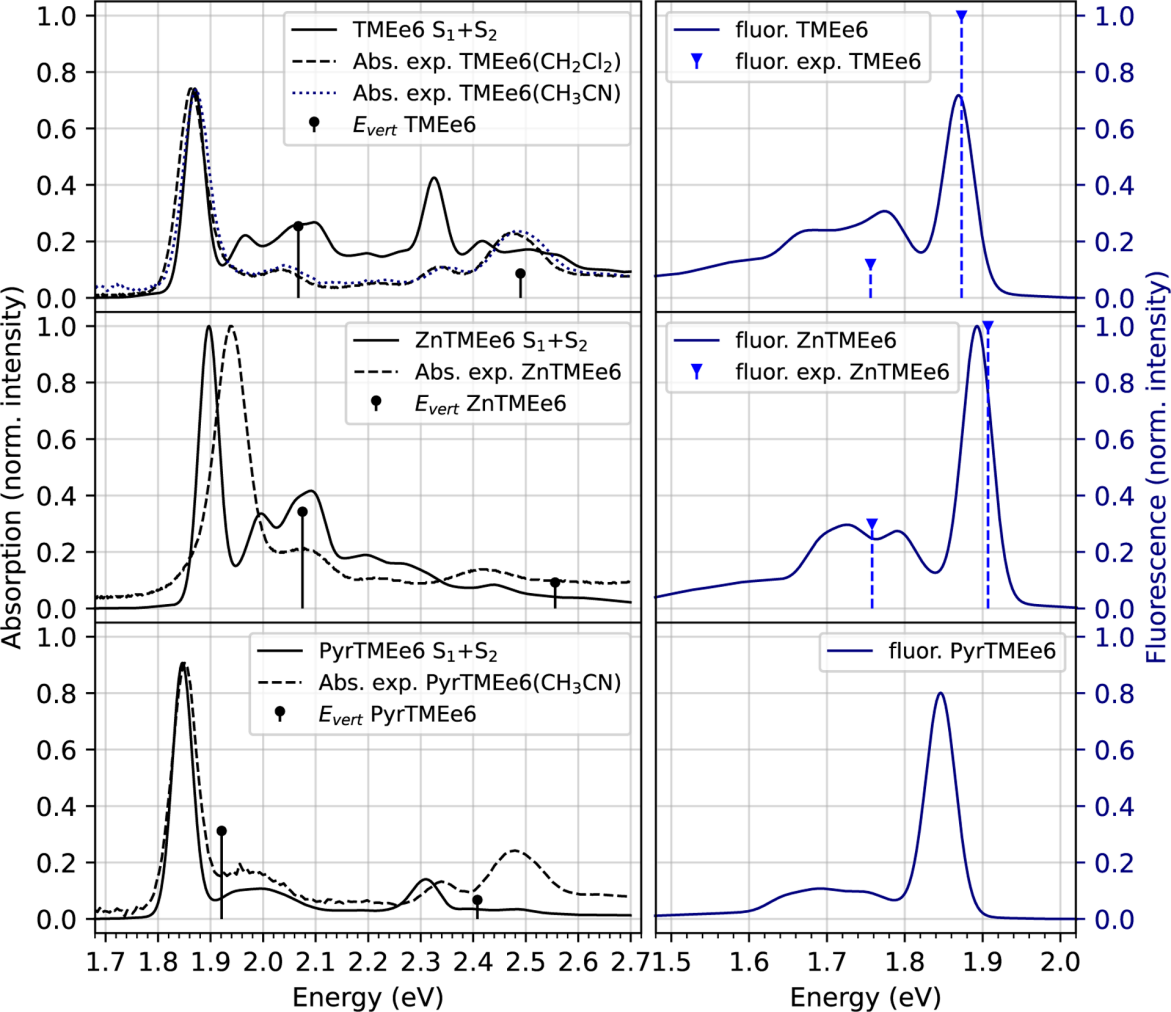
Calculated absorption (left) and fluorescence spectra (right) of free (top)/zinc­(center)/pyridine (bottom) chlorin e6 trimethyl-ester derivatives in CH_2_Cl_2_ (solid lines) and their experimental data (dotted lines).^13,32,33^ Calculated absorption spectra (full-CAM-B3LYP/def2-SVP) were normalized at the ratio between S_1_ oscillator strengths at S0 optimized geometries, while fluorescence spectra were normalized at the ratio between S_1_ oscillator strengths at S_1_ optimized geometries. For reference, VEEs calculated at full-CAM-B3LYP/def2-SVP are shown as vertical solid lines, scaled to the oscillator strengths of each transition. Experimental maximum signals of fluorescence are shown as vertical dotted lines in blue. No additional shifts were made to the spectra.

The experimental spectra of TMEe6 and ZnTMEe6 differ in a higher molar absorptivity of the Q_*y*_ band and the vanishing of the Q_*x*_ band in ZnTMEe6, which is related to an orbital degeneracy from an increased symmetry.^14,63^ This effect is usually used to confirm metal chelation, and our results reproduce it. The experimental fluorescence spectra exhibit an intense band at the same position as the Q_*y*_ absorption band, along with a weak shoulder band attributed to vibrational effects. These molecules are usually nonphosphorescent at room temperature, although some studies have reported a phosphorescence spectrum at 77 K.^65^ The calculated phosphorescence rates support the absence of phosphorescence spectra at room temperature. The calculated spectra are in agreement with the experimental ones, however, there are some differences. Below, the differences between full-TDDFT calculated spectra and experimental data will be discussed.

The calculated Q_*y*_(0,0) absorption band for TMEe6, located at 1.87 eV, is blueshifted by 0.01 eV in comparison to the experimental spectrum in CH_3_CN. The vertical excitation energy (VEE) for this transition is blue-shifted by 0.18 eV, while its adiabatic energy is blueshifted by 0.03 eV. Experimentally, the Q_*y*_ band is accompanied by a vibrational band at 2.04 eV. However, the calculated spectrum shows two vibrational bands at 1.97 and 2.10 eV. If we compare the experimental band with the more intense calculated vibrational band (2.10 eV), it is blueshifted by 0.06 eV.

The experimental Q_*x*_(0,0) band for TMEe6 exhibits a low-intensity band at 2.34 eV (530 nm) and a more intense vibrational band at 2.48 eV (500 nm).^32,33,64^ The band at 530 nm is associated with the Q_*x*_(0–0) band, taking into account the results of Zenkevich and collaborators.^65^ The calculated spectrum for this transition exhibits an intense (0–0) band at 2.32 eV, accompanied by a redshift of just 0.01 eV, and two vibrational bands at 2.42 and 2.51 eV. If we compare the third band (2.51 eV), as was done for the Q_*y*_ vibrational band, it is blueshifted by 0.03 eV. The intensities of the calculated Q_*x*_ spectrum differ from those of the experimental spectrum, with the (0–0) transition being less intense than its vibrational band in the experimental spectrum.

For ZnTMEe6, the calculated Q_*y*_(0,0) band at 1.90 eV is red-shifted by 0.05 eV in comparison to the experimental band in CH_2_Cl_2_.^32,33^ The VEE for this transition is blueshifted by 0.14 eV, while its adiabatic energy is blue-shifted by 0.01 eV. The vibrational band associated with this transition is experimentally located at 2.08 eV, while the calculated ones are located at 2.00 and 2.09 eV. If we compare the most intense calculated vibrational band (2.09 eV), as was done for TMEe6, this calculated band is blueshifted by 0.01 eV.

For ZnTMEe6, the experimental Q_*x*_ band vanishes; however, a very weak band is observed at 2.43 eV in the literature spectrum for ZnTMEe6.^33^ The calculation of the S_2_ absorption spectrum was not possible within the AH model due to problems within the harmonic approximation and the calculated displacement vector; therefore, the VG model was used to compute it. The calculated Q_*x*_(0,0) band is located at 2.44 eV, being blueshifted by 0.01 eV, compared to the experimental data. Its VEE is blueshifted by 0.13 eV, and its adiabatic energy is red-shifted by 0.03 eV.

The calculated absorption spectrum for PyrTMEe6 displays a Q_*y*_(0–0) band at 1.85 eV, which meets the experimental band in CH_3_CN.^34^ Its VEE is blueshifted by 0.07 eV, while its adiabatic energy is blueshifted by 0.01 eV. There is a vibrational band at 2.00 eV, which has less intensity than those calculated for TMEe6 and ZnTMEe6, and agrees with the experimental vibrational band that spans the same region.

Experimental Q_*x*_ bands for PyrTMEe6 are very similar to TMEe6 Q_*x*_ bands, with two maxima at 2.34 and 2.48 eV, being the latter more intense. The calculated Q_*x*_(0–0) band is located at 2.30 eV and it is red-shifted by 0.04 eV compared to the experimental data. Additionally, its VEE is blueshifted by 0.06 eV while its adiabatic energy is blueshifted by only 0.03 eV. There are two calculated vibrational bands associated with the Q_*x*_ band, located at 2.39 and 2.49 eV. If we compare the third band (2.49 eV), similarly to what was done for the Q_*x*_ band in TMEe6, it is blueshifted by 0.01 eV.

The calculated fluorescence spectrum for TMEe6 shows a maximum of the emission band at 1.87 eV (663 nm), which is very close to the reported one at 668 nm for chlorine e6 in ethanol,^65^ as well as to the 663 nm for o-QM-TMEe6 derivatives in DMF.^13^ In the case of ZnTMEe6, the calculated fluorescence band maximum is observed at 1.89 eV (654 nm), which is close to the reported value of 650 nm for ZnTMEe6 in DMF.^13^ The solvent is different for the experimental and calculated spectra; however, solvent effects are expected to be small, as it was previously shown for pyro-pheophorbide a.^10^ In both molecules, fluorescence spectra exhibit a vibrational band between 1.7 eV (730 nm) and 1.8 eV (689 nm), which has been reported experimentally as a shoulder band at 705 nm.^13^ As for TMEe6 and ZnTMee6, PyrTMEe6 shows a maximum fluorescence band at 1.85 eV, which is a specular image of its absorption spectrum.

Herzberg–Teller contributions to the calculated spectra are show in Table S4 in the Supporting Information. In general HT effects are small and modulate the intensity of the vibrational bands, but do not alter the general form of the calculated spectra.

Both calculated absorption and fluorescence spectra follow the same trends in the derivatives studied. In comparison with TMEe6, the ZnTMEe6 spectrum is blueshifted by 0.03 eV, and the PyrTMEe6 spectrum is red-shifted by 0.03 eV. The calculated bands (0–0) agree very well with the experimental spectra without additional shifts; however, associated vibrational bands exhibit some differences, including the presence of an additional band, both for Q_*y*_ and Q_*x*_ vibrational bands. Additionally, calculated Q_*x*_ bands exhibit differences in their intensities, with the (0–0) transition being less intense for experimental spectra. This issue may be related to the anharmonicity of certain vibrational modes, which the harmonic approximation cannot accurately describe.^56,73^ However, our calculations cannot explain the relatively small intensity of the Q_*x*_(0–0) experimental bands.

We also tested the impact of conformer selection on the calculated spectra. To test this, we performed an optimization and subsequent spectral calculations of different conformers of TMEe6 for their absorption and fluorescence processes. Their structures are shown in Figure S16 and their spectra in Figures S17–S18 in the Supporting Information. The spectra of these different conformers show small displacements of Q_*y*_ bands less than 0.02 eV to the blue. While these displacements may result in slight broadening of the spectrum, no discernible differences are apparent in the bands of maximum absorption. The more relevant effects expected for the conformer selection are those in the vibrational bands. As shown in Figure S17, there is a slight change in their relative intensities. However, their vibrational bands are essentially the same, except for the band at 2.06 eV of CONF84, which shows more intensity compared to the other conformers analyzed. The discrepancies observed between the experimental Q_*x*_ vibrational band at 2.48 eV and the calculated spectra are not attributable to conformer selection, as this region remains unchanged regardless of the conformer selection. It is reasonable to conclude that the effect of conformers on the calculated spectra can be overlooked, given that the variations are so minor as to be negligible. However, is essential to note that the method employed is heavily dependent on the harmonic approximation and the Hessians calculated at the TDDFT level, which can significantly alter the vibronic structure of the calculated spectrum. Furthermore, as it was described in the methodology, it was required to exclude vibrational frequencies below 350 cm^–1^. This accumulation of approximations of the method may explain the main differences between the calculated vibrational bands and the experimental spectra.

Phosphorescence spectra are crucial for the study of photosensitizers, as they allow for the experimental measurement of triplet state energies.^65^ Although these molecules do not exhibit phosphorescence at room temperature, the phosphorescence spectrum could be measured at 77 K, as was done before for chlorin e6.^65^ The calculated phosphorescence spectra at 298 K and 0 K (sticks) are shown in Figures S13–S15 in the Supporting Information. Unfortunately, the experimental phosphorescence spectra for TMEe6, ZnTMEe6, or PyrTMEe6 have not been reported in the literature. However, supposing that Chlorin e6 shares spectroscopic properties with TMEe6, it is possible to compare the calculated phosphorescence spectra with the experimental one at 77 K for Chlorin e6,^65^ showing a redshift of 0.2 eV with respect to the experimental. This redshift would suggest that triplet energies are underestimated by TDA/TDDFT, as was reported for CAM-B3LYP.^42,71^

### 3.5 Adiabatic Energy Diagrams

The adiabatic energies and their ZPE corrections (in parentheses) of each excited state relative to their ground state S_0_ for TMEe6, ZnTMEe6, and PyrTMEe6 are shown in Figure 5. Additional vertical energy diagrams at the optimized geometry of each electronic state are shown in the Supporting Information (Figures S1–S3).

**Figure 5:**
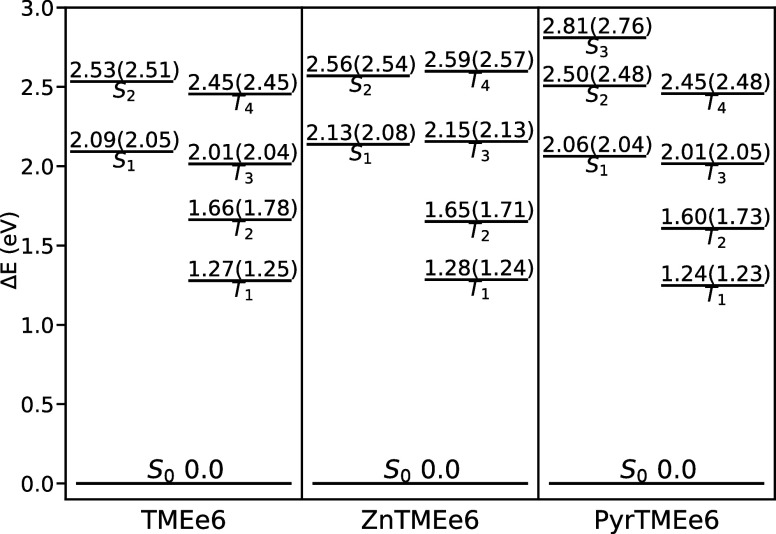
Adiabatic energies of optimized excited states relative to their ground state S_0_. Energies were computed in CH_2_Cl_2_ at TDA/CAM-B3LYP­(CPCM)/def2-SVP level of theory. ZPE corrections are shown between parentheses.

Following Kasha’s rule^53^ and based on Figure 5 it is possible to suggest ISC channels between S_1_ ⇝ {T_1_, T_2_, T_3_}. The T_3_ state for ZnTMEe6 is slightly above the S_1_, but is thermally accessible at room temperature. An analysis of this adiabatic energy diagram reveals that zinc has a negligible effect on the energies of the T_1_ and T_2_ states. However, it has a destabilizing effect on the T_3_ and T_4_ states, with energies of approximately 0.10 eV higher than free base TMEe6. Similarly, energies of the S_1_ and S_2_ states are increased to a lesser extent by approximately 0.03 eV. This higher energy makes the adiabatic energy gap between the S_1_–T_3_ states smaller, making them almost isoenergetic, which can potentially enhance ISC. However, this leads to a higher S_1_ ⇝ {T_1_, T_2_} energy gap, which could diminish ISC rates for those channels.

As mentioned above, the addition of pyridine results in the formation of a new S_3_ state of charge transfer (CT) character between the chlorin macrocycle and the pyridine aromatic ring, with an energy of 2.81 eV. Furthermore, pyridine has a stabilizing effect on the S_1_, S_2_, T_1_ and T_2_ states, lowering their energies by around 0.05 eV. This is a relatively minor effect, but it is observed as a redshift in the absorption bands.

### 3.6 Calculated Transition Rate Constants

#### 3.6.1 Fluorescence Rate Constants

Table 4 shows the calculated fluorescence rate constants for TMEe6, ZnTMEe6, and PyrTMEe6. In literature, there are reported fluorescence rate constants for *o*-QM-TMEe6 in DMF,^13^ chlorin e6 and chlorin p6 trimethyl ester in ethanol,^65^ and ZnTMEe6 in DMF.^13^ To the best of our knowledge, there are no reported fluorescence rate constants for TMEe6 and PyrTMEe6. However, we expect TMEe6 to exhibit fluorescence similar to chlorin e6. These data have been used for comparison with the calculated ones.

**Table 4:** Fluorescence Rate Constants Calculated with the Path Integral Approach from CAM-B3LYP/def2-SVP Energies and Geometries^a^

molecule	*E*_AD_ (eV)	λ_F_(nm)	*k*_F_^FCHT^(s^–1^)	%HT	*k*_F_^FC^(s^–1^)
TMEe6 (full-TDDFT)	1.90	664	1.44 × 10^8^	26.14	1.06 × 10^8^
TMEe6 (TDA/TDDFT)	2.09	602	2.08 × 10^8^	27.07	1.52 × 10^8^
exp. Chlorin e6 in ethanol^65^	668	2.27 × 10^8^			
exp. *o*-QM-TMEe6 in DMF^13^	664	2.85 × 10^8^			
ZnTMEe6 (full-TDDFT)	1.95	655	1.90 × 10^8^	25.82	1.41 × 10^8^
ZnTMEe6 (TDA/TDDFT)	2.14	595	2.79 × 10^8^	27.40	2.02 × 10^8^
exp. ZnTMEe6 in DMF^13^	650	3.20 × 10^8^			
PyrTMEe6 (full-TDDFT)	1.87	672	1.59 × 10^8^	14.69	1.36 × 10^8^
PyrTMEe6 (TDA/TDDFT)	2.06	607	2.21 × 10^8^	15.40	1.87 × 10^8^

a *E*_AD_ is the adiabatic energy difference in eV between ground state S_0_ and the first singlet S_1_ calculated at S_1_ minimum. λ_F_(nm) corresponds to emission maxima from calculated spectrum. *k*_F_^FCHT^ is the transition rate constant calculated with Herzberg–Teller contributions, %HT is the Herzberg–Teller contribution to calculated rate constant, and *k*_F_^FC^ is the transition rate constant calculated by employing direct Franck–Condon approximation.

The computed *k*_F_ rates are of the same order of magnitude as the experimental rate constants reported in the literature, which are approximately 10^8^ s^–1^. Zinc addition appears to enhance the fluorescence process by approximately 30–40%, as supported by both calculated and experimental data. On the other hand, pyridine addition does not notably affect the calculated rates, which are just around 10% higher. Herzberg–Teller contribution to fluorescence rates is not greater than 27%. Zinc addition does not affect these contributions, but pyridine addition decreases them to approximately 15%. A comparison between the calculated and experimental fluorescence rate constants shows that the calculated rate constants are underestimated by around 40–50% when full-TDDFT is employed, and 10% when TDA/TDDFT is employed. Additionally, a comparison between *k*_F_^FC^ and *k*_F_^FCHT^ infers that HT effects are required to compute a fluorescence rate constant closer to experimental values. These errors are less than the expected ranges between 50 and 120% that other authors have reported using a similar methodology.^29,74^ We recall that the experimental error associated with these rates can also be large.^53,74^

Calculated band maxima (λ_F_) match experimental values when full-TDDFT energies are employed. If TDA is used, the rates are in a better agreement with the experimental values; however, the spectra associated with them are blueshifted by 0.2 eV with respect to the experimental data, as reported by other authors.^74^ This effect appears to be related to higher S_0_ → S_1_ excitation energies using TDA which enters eq 1 cubically.

#### 3.6.2 Phosphorescence Rate Constants

The calculated phosphorescence rate constants, *k*_P_, are shown in Table 5. It is important to note that these rates are 8 orders of magnitude smaller than those for fluorescence and ISC. This is consistent with the fact that these molecules do not exhibit phosphorescence at room temperature. Their Herzberg–Teller contributions confirm that this process is mainly promoted by vibrational effects, as expected for a spin-forbidden transition. The addition of zinc and pyridine results in a slight but similar increase in the phosphorescence rate. However, zinc addition decreases their HT contributions, suggesting that other effects are at play. This diminution can be attributed to the heavy-atom effect of zinc, being an atom from the fourth row, though it is not strong enough to increase the calculated SOCMEs notably (see Tables S6–S8).

**Table 5:** TDA/TDDFT/CAM-B3LYP­(CPCM)/def2-SVP Phosphorescence Rate Constants (T_1_ → S_0_) for Chlorin e6 Trimethyl Ester (TMEe6) and Their ZnTMEe6 and PyrTMEe6 Derivatives in CH_2_Cl_2_

molecule	channel^a^	*k*_P_ ^b^ (s^–1^)	HT cont.^c^ (%)
TMEe6	T_1_ → S_0_	1.75	90.33
ZnTMEe6	T_1_ → S_0_	8.09	57.90
PyrTMEe6	T_1_ → S_0_	8.91	90.34

a Each channel has three sublevels due to its multiplicity.

b As the arithmetic mean of sublevels.

c As the weighted arithmetic mean based on its *k*_P_. Details for sublevels are shown in Table S5 in Supporitng Information.

#### 3.6.3 Intersystem Crossing Rate Constants

A suitable photosensitizer, must efficiently populate its triplet states from the S_1_ excited state and have absorption in the desired region. This generally occurs due to a spin–orbit coupling (SOC) that promotes intersystem crossing, a small energy gap between singlet and triplet states (Δ*E*_S–T_) that promotes a horizontal transition between vibrational-electronic states, and a change in angular momentum *L* that balances the change in spin momentum *S* in order to preserve total angular moment *J* (*J* = *L* + *S*). This change in angular momentum is seen as a transition that involves orbitals of different character.^53^

Organic dyes, like TMEe6, have an efficient ISC with triplet quantum yields of around 0.65.^7,65^ However, they do not satisfy some of the general characteristics that define a good photosensitizer. The orbitals involved in the transitions of low-lying excited states are of (π, π*) character, which is reflected in small SOCMEs, less than 1 cm^–1^. Therefore, direct SOC is not the main promoter of ISC. Thus, taking into account vibrational effects is necessary to explain the experimental ISC yields for these molecules, as has been observed in previous porphyrin studies.^75,76^ The calculated intersystem crossing rate constants, *k*_ISC_, are shown in Table 6.

**Table 6:** Intersystem Crossings Results between the First Singlet State and Triplets States below S_1_ Energy of Chlorin e6 Trimethyl-Ester Derivatives in CH_2_Cl_2_

molecule	channel^a^	Δ*E*_S–T_ (eV)	*k*_ISC_ ^b^ (s^–1^)	HT^c^(%)	Σ*k*_ISC_ ^d^ (s^–1^)
TMEe6	S_1_ ⇝T_1_	0.814	1.22 × 10^7^	64.07	1.29 × 10^8^
	S_1_ ⇝T_2_	0.429	1.13 × 10^8^	38.34	
	S_1_ ⇝T_3_	0.078	4.25 × 10^6^	80.93	
ZnTMEe6	S_1_ ⇝T_1_	0.853	7.46 × 10^6^	44.68	2.83 × 10^8^
	S_1_ ⇝T_2_	0.486	2.14 × 10^7^	65.11	
	S_1_ ⇝T_3_	–0.018	2.54 × 10^8^	96.83	
PyrTMEe6	S_1_ ⇝T_1_	0.814	6.46 × 10^7^	76.36	1.82 × 10^8^
	S_1_ ⇝T_2_	0.455	9.93 × 10^7^	53.74	
	S_1_ ⇝T_3_	0.047	1.84 × 10^7^	66.84	

a Each channel has three sublevels due to their multiplicity.

b As the sum of *k*_ISC_ for each sublevel.

c As the weighted arithmetic mean of HT contributions of each sub level.

d As the sum of rates for each channel. Details for sublevels are shown in Tables S6–S8 in Supporting Information.

The calculated transition rates for ISC show that the main deactivation channel is S_1_ ⇝ T_2_ for both TMEe6 and PyrTMEe6. In contrast, for ZnTMEe6, the dominant ISC channel is S_1_ ⇝ T_3_. It should be noted that HT effects are dominant in all ISC channels. This observation supports the importance of including HT effects in cases where calculated SOCMEs are small, such as in organic chromophores.

Comparing PyrTMEe6 and TMEe6 ISC rates, pyridine addition enhances the S_1_ ⇝ T_1_ channel by a factor of 5.3, and the S_1_ ⇝ T_3_ channel by 4.3, whereas it reduces the S_1_ ⇝ T_2_ channel by 10%, yielding an ISC rate constant of approximately 1 × 10^8^ s^–1^. This combination of effects enhances the total ISC rate by around 41%.

In the case of ZnTMEe6 ISC channels, the S_1_ ⇝ T_1_ and S_1_ ⇝ T_2_ rates decrease by around 1 order of magnitude, which is consistent with the increase in the energy gap between these states. This increase also favors the channel S_1_ ⇝ T_3_ by shortening the energy gap between these states. Even though T_3_ is higher in energy than S_1_, the S_1_–T_3_ energy gap is lower than thermal energy at room temperature (*k*_B_ *T* = 0.025 eV), favoring the horizontal transition. This channel exhibits a higher SOCME and a noteworthy contribution of 96% of Herzberg–Teller effects to the calculated ISC rate constant.

It is interesting to examine the modes with the highest SOCME derivatives because of the significance of HT contributions to the ISC rates shown in Table 6. The derivatives of the SOCMEs for each normal mode are provided in the Supporting Information. It is notable that no specific vibrational modes stand out based on the SOCME derivatives, as all are under 10^–3^ cm^–1^. However, with approximately 250 vibrational modes, it is the combined effect of these small contributions that ultimately has a noticeable influence on the calculated ISC rates.

We also tested the effect of varying the adiabatic energy difference between the singlet and triplet states, Δ*E*(S–T), by ± 0.1 eV in the calculations of the *k*_ISC_. These results are shown in Tables S9–S11 and Figures S19–S21. These results show that when Δ*E*(S–T) becomes negative, the calculated *k*_ISC_ drops by 2 orders of magnitude, as seen in the S_1_ ⇝ T_3_ transition for TMEe6 and PyrTMEe6. This can be interpreted as the triplet state increasing in energy relative to the singlet state, making it less accessible. This is not observed for ZnTMEe6. The ISC transition S_1_ ⇝ T_2_ for PyrTMEe6 (Figure S21) doubles when the Δ*E*(S–T) lowers by 0.1 eV, but stay in the same order of magnitude. For the other transitions, the calculated *k*_ISC_ remains in the same order of magnitude.

That said, the efficiency of a PS to populate a triplet state not only relies on its *k*_ISC_, but also on the balance between other competing deactivation channels that can occur from its S_1_ excited state. Other possible channels are fluorescence and internal conversion. By neglecting internal conversion (IC), only fluorescence competes against ISC. The calculated ISC rates are of the same order of magnitude as the calculated fluorescence rates, replicating the experimental competitiveness of these processes. However, the calculated fluorescence rates are higher than the ISC rates, contrary to what the experimental quantum yields of TMEe6 suggest. This deviation from experimental quantum yields might be due to different factors: the use of TDDFT energies, the harmonic approximation for all vibrational modes, and the quality of the Hessian computed by CAM-B3LYP/def2-SVP.

## 4 Conclusions

In this article we presented a computational study of absorption, fluorescence, phosphorescence, and ISC processes of free-base chlorin e6 trimethyl ester (TMEe6), ZnTMEe6, and PyrTMEe6 derivatives using a methodology based on the combination of a conformational exploration, and the Fermi’s golden rule derived path integral formalism, implemented within time-dependent density functional theory (TD-DFT). Molecular orbital contributions to the excited states obtained with CAM-B3LYP/def2-SVP agree with the Gouterman four-orbital model, which is usually used to explain chlorins spectra. The calculated spectra and transition rates are reasonably close to the literature data, replicating vibrational bands, with deviations of less than 0.05 eV for absorption and fluorescence bands when full-TDDFT is used, and an overestimation of 0.2 eV when TDA/TDDFT is used. However, when employing full-TDDFT, the calculated fluorescence rates are underestimated by approximately 40–50%; this underestimation is reduced to 10% when TDA is used. There are some differences in vibrational bands, where an additional band with no experimental counterpart appears, and the intensity from the calculated Q_*x*_ bands does not match experimental intensities. Phosphorescence spectra exhibit a redshift of approximately 0.2 eV, which is attributable to the energy underestimation of triplets using TDA/CAM-B3LYP.^42^ The calculated fluorescence and ISC rates are in the order of 10^8^ s^–1^, and phosphorescence rates in the ∼10° s^–1^ in agreement with the available experimental data. Despite the underestimation of fluorescence rates, they replicate chemical trends expected for the explored derivatives, given that the experimental error in these rates can also be large.^29,53,55,74^ The Herzberg–Teller effects with contributions between 38–96% for ISC, 15–27% for fluorescence and 57–90% for phosphorescence, show the importance of including vibrational effects on the studied processes. Additionally, the effect on the selection of the minimum energy conformer is negligible. These results show valuable insights into the photophysical processes of TMEe6, ZnTMEe6, and PyrTMEe6: they give a good description of electronic structure and spectra, calculated rates are in the same order of magnitude as experimental data available, and they are in the same error margin of 50% that other studies of similar methods report.^29,55,74^

## Supplementary Material







## Notes

The ESI contains optimized geometries (as separated files), excitation energies, vibronic spectra, conformer effects, SOCME derivatives (as separated files) and transition rate constants for TMEe6, ZnTMEe6, and PyrTMEe6 (PDF)
Optimized_geometries.zip: optimized geometries of basal and excited states for TMEe6, ZnTMEe6, and PyrTMEe6
SOCME_derivatives.zip: contains the files ISC_TMEe6_dSOCME.xls, ISC_ZnTMEe6_ (ZIP)
dSOCME.xls and ISC_PyrTMEe6_dSOCME.xls, with the SOCME derivatives for each vibrational mode for each spin sublevel for each computed transition (ZIP)
