# Supplementary material for: Photophysical Properties of Free, Zinc‑, and Pyridine- Chlorin e6 Trimethyl Ester: A Computational Study with Vibrational Effects
Source: ACS Omega. 2026 Mar 24;11(13):20241–53. doi: 10.1021/acsomega.5c10268 (PMC13063191; doi:10.1021/acsomega.5c10268)
Supplement: Supplementary file 1 [file ao5c10268_si_001.pdf]

# Electronic Supplementary Information for Photophysical properties of free, zinc-, and pyridine- chlorin e6 trimethyl ester: A computational study with vibrational effects

Marlon D. Suárez Ruiz, Martha C. Daza, and Markus Doerr*

## Contents

**A Optimized geometries** 4

**B Absolute energies calculated at the stationary points** 5

**C Energy diagrams** 6

**D Calculated spectra with vibronic progression** 9

- **D**.1 Absorption spectra . . . 9
  - **D**.1.1 Herzberg-Teller contributions to the calculated absorption spectra . . . 11
- **D**.2 Fluorescence spectra . . . 12
- **D**.3 Phosphorescence spectra . . . 14
- **D**.4 Effect of the selection of conformer . . . 15

**E Calculated transition rate constants** 16

- **E**.1 Phosphorescence rate constants . . . 16
- **E**.2 Intersystem crossing rate constants . . . 16
  - **E**.2.1 Effect of the energy gap $\Delta E(S\text{-}T)$ on the intersystem crossing rate constants $k_{ISC}$ . . . 17

## List of Figures

S1 Energy diagram for adiabatic energies of each stationary point for TMEe6. The energies were calculated using TDA/CAM-B3LYP/def2-SVP. . . . . 7
S2 Energy diagram for adiabatic energies of each stationary point for ZnTMEe6. The energies were calculated using TDA/CAM-B3LYP/def2-SVP. . . . . 7
S3 Energy diagram for adiabatic energies of each stationary point for PyrTMEe6. The energies were calculated using TDA/CAM-B3LYP/def2-SVP. . . . . 8
S4 Calculated absorption spectrum of TMEe6 for $S_1$ transitions. Adiabatic and vertical energies calculated with CAM-B3LYP/def2-SVP are shown as vertical lines. The experimental spectrum is shown as dotted lines, taken from Blanco Ramírez and Calderón Hernández [1]. . . . . 9
S5 Calculated absorption spectrum of ZnTMEe6 for $S_1$ transitions. Adiabatic and vertical energies calculated with CAM-B3LYP/def2-SVP are shown as vertical lines. The experimental spectrum is shown as dotted lines, taken from Muñoz [2]. . . . . 9
S6 Calculated absorption spectrum of PyrTMEe6 for $S_1$ transitions. Adiabatic and vertical energies calculated with CAM-B3LYP/def2-SVP are shown as vertical lines. The experimental spectrum is shown as dotted lines, taken from Tarazona Cáceres [3]. . . . . 10
S7 Calculated absorption spectrum of TMEe6 for $S_2$ transitions. Adiabatic and vertical energies calculated with CAM-B3LYP/def2-SVP are shown as vertical lines. The experimental spectrum is shown as dotted lines, taken from Blanco Ramírez and Calderón Hernández [1]. . . . . 10
S8 Calculated absorption spectrum of ZnTMEe6 for $S_2$ transitions. Adiabatic and vertical energies calculated with CAM-B3LYP/def2-SVP are shown as vertical lines. The experimental spectrum is shown as dotted lines, taken from Muñoz [2]. . . . . 10
S9 Calculated absorption spectrum of PyrTMEe6 for $S_2$ transitions. The experimental spectrum is shown as dotted lines. Adiabatic and vertical energies calculated with CAM-B3LYP/def2-SVP are shown as vertical lines. Experimental spectrum taken from Tarazona Cáceres [3]. . . . . 11
S10 Calculated fluorescence spectrum of TMEe6. Adiabatic and vertical energies calculated with CAM-B3LYP/def2-SVP are shown as vertical lines. . . . . 13
S11 Calculated fluorescence spectrum of ZnTMEe6. Adiabatic and vertical energies calculated with CAM-B3LYP/def2-SVP are shown as vertical lines. . . . . 13
S12 Calculated fluorescence spectrum of PyrTMEe6. Adiabatic and vertical energies calculated with CAM-B3LYP/def2-SVP are shown as vertical lines. . . . . 13
S13 Calculated phosphorescence spectrum of TMEe6, normalized over the mean of the spectra of each sublevel. . . . . 14
S14 Calculated phosphorescence spectrum of ZnTMEe6, normalized over the mean of the spectra of each sublevel. . . . 14
S15 Calculated phosphorescence spectrum of PyrTMEe6, normalized over the mean of the spectra of each sublevel. . . . 14
S16 Geometries of the chosen conformers to test their effect on the absorption spectra of TMEe6 and their respective Boltzmann weights (%W). . . . . 15
S17 Calculated absorption spectra for the $Q_y$ band, $S_0 \rightarrow S_1$ and the $Q_x$ band, $S_0 \rightarrow S_2$. . . . . 15
S18 Calculated fluorescence spectra of the chosen conformers. . . . . 16
S19 Intersystem crossing rate constants for TMEe6, calculated with various singlet-triplet energy differences ΔE(S-T), for each one of the $S_1 \rightsquigarrow \{T_1, T_2, T_3\}$ transitions. The bar chart to the right employs a logarithmic scale. . . . . 18
S20 Intersystem crossing rate constants of ZnTMEe6, calculated with various singlet-triplet energy differences ΔE(S-T), for each of the $S_1 \rightsquigarrow \{T_1, T_2, T_3\}$ transitions. The bar chart to the right employs a logarithmic scale. . . . . 19
S21 Intersystem crossing rate constants of PyrTMEe6, calculated with various singlet-triplet energy differences ΔE(S-T), for each of the $S_1 \rightsquigarrow \{T_1, T_2, T_3\}$ transitions. The bar chart to the right employs a logarithmic scale. . . . . 20

## List of Tables

S1 Relative energies calculated at the stationary points for the basal state and each excited state of TMEe6. The energies were calculated using CAM-B3LYP/def2-SVP. . . . . 5
S2 Relative energies calculated at the stationary point for the basal state and each excited state for ZnTMEe6. The energies were calculated using CAM-B3LYP/def2-SVP. . . . . 5
S3 Relative energies calculated at the stationary point for the basal state and each excited state for PyrTMEe6. The energies were calculated using CAM-B3LYP/def2-SVP. . . . . 6
S4 Herzberg-Teller contributions to the calculated absorption spectra. . . . . 12
S5 Phosphorescence rate constants with sublevel detail. . . . . 16
S6 Intersystem crossing rate constants for TMEe6 with sublevel detail. . . . . 17
S7 Intersystem crossing rate constants for ZnTMEe6 with sublevel detail. . . . . 17

S8 Intersystem crossing rate constants for PyrTMEe6 with sublevel detail. . . . . . . . . . . . . . . . . . . . . . . . . . . 17
S9 Intersystem crossing rate constants of TMEe6, calculated with various singlet-triplet energy differences ΔE(S-T). . . 17
S10 Intersystem crossing rate constants of ZnTMEe6, calculated with various singlet-triplet energy differences ΔE(S-T). . 18
S11 Intersystem crossing rate constants of PyrTMEe6, calculated with various singlet-triplet energy differences ΔE(S-T). 19

## A Optimized geometries

In this section we present a list of the files of optimized geometries calculated with TD-DFT CAM-B3LYP/def2-SVP for ground and excited states of the chlorin e6 trimethyl ester (TMEe6) and its derivatives with zinc (ZnTMEe6) and with pyridine (PyrTMEe6). Triplet states have been optimized using the Tamm-Dancoff approximation (TDA).

**S1**. Chlorin e6 trimethyl ester (TMEe6):

(**S1.1**) Optimized ground state, $S_0$ File: OPT_GEOMETRIES/TMEe6/optS0.xyz
(**S1.2**) Optimized first singlet state, $S_1$ File: OPT_GEOMETRIES/TMEe6/optS1.xyz
(**S1.3**) Optimized second singlet state, $S_2$ File: OPT_GEOMETRIES/TMEe6/optS2.xyz
(**S1.4**) Optimized third singlet state, $S_3$ File: OPT_GEOMETRIES/TMEe6/optS3.xyz
(**S1.5**) Optimized first triplet state, $T_1$ File: OPT_GEOMETRIES/TMEe6/optT1.xyz
(**S1.6**) Optimized second triplet state, $T_2$ File: OPT_GEOMETRIES/TMEe6/optT2.xyz
(**S1.7**) Optimized third triplet state, $T_3$ File: OPT_GEOMETRIES/TMEe6/optT3.xyz
(**S1.8**) Optimized fourth triplet state, $T_4$ File: OPT_GEOMETRIES/TMEe6/optT4.xyz

**S2**. Zinc chlorin e6 trimethyl ester (ZnTMEe6):

(**S2.1**) Optimized ground state, $S_0$ OPT_GEOMETRIES/ZnTMEe6/optS0.xyz
(**S2.2**) Optimized first singlet state, $S_1$ File: OPT_GEOMETRIES/ZnTMEe6/optS1.xyz
(**S2.3**) Optimized second singlet state, $S_2$ File: OPT_GEOMETRIES/ZnTMEe6/optS2.xyz
(**S2.4**) Optimized third singlet state, $S_3$ File: OPT_GEOMETRIES/ZnTMEe6/optS3.xyz
(**S2.5**) Optimized first triplet state, $T_1$ File: OPT_GEOMETRIES/ZnTMEe6/optT1.xyz
(**S2.6**) Optimized second triplet state, $T_2$ File: OPT_GEOMETRIES/ZnTMEe6/optT2.xyz
(**S2.7**) Optimized third triplet state, $T_3$ File: OPT_GEOMETRIES/ZnTMEe6/optT3.xyz
(**S2.8**) Optimized fourth triplet state, $T_4$ File: OPT_GEOMETRIES/ZnTMEe6/optT4.xyz

**S3**. Pyridine chlorin e6 trimethyl ester (PyrTMEe6):

(**S3.1**) Optimized first singlet state, $S_1$ File: OPT_GEOMETRIES/PyrTMEe6/optS0.xyz
(**S3.2**) Optimized second singlet state, $S_2$ File: OPT_GEOMETRIES/PyrTMEe6/optS1.xyz
(**S3.3**) Optimized third singlet state, $S_3$ File: OPT_GEOMETRIES/PyrTMEe6/optS2.xyz
(**S3.4**) Optimized fourth singlet state, $S_4$ File: OPT_GEOMETRIES/PyrTMEe6/optS3.xyz
(**S3.5**) Optimized first triplet state, $T_1$ File: OPT_GEOMETRIES/PyrTMEe6/optS4.xyz
(**S3.6**) Optimized second triplet state, $T_2$ File: OPT_GEOMETRIES/PyrTMEe6/optT1.xyz
(**S3.7**) Optimized third triplet state, $T_3$ File: OPT_GEOMETRIES/PyrTMEe6/optT2.xyz
(**S3.8**) Optimized second triplet state, $T_2$ File: OPT_GEOMETRIES/PyrTMEe6/optT3.xyz

## B Absolute energies calculated at the stationary points

In this section, relative energies from single-point calculations at the CAM-B3LYP/def2-SVP level of theory at the stationary points presented in section A are shown for each of the investigated molecules.

**Table S1** Relative energies calculated at the stationary points for the basal state and each excited state of TMEe6. The energies were calculated using CAM-B3LYP/def2-SVP.

| | @S0 | @S1 | @S2 | @S3 | @T1 | @T2 | @T3 | @T4 |
|---|---|---|---|---|---|---|---|---|
| State | Relative energies of excited states full TDDFT | | | | | | | |
| S1 | 2.067 | 1.830 | 1.861 | 1.933 | 1.766 | 1.777 | 1.965 | 1.997 |
| S2 | 2.490 | 2.338 | 2.218 | 2.411 | 2.339 | 2.306 | 2.432 | 2.421 |
| S3 | 3.149 | 3.084 | 3.195 | 3.015 | 3.097 | 3.114 | 3.024 | 3.007 |
| T1 | 0.919 | 0.665 | 0.257 | 0.929 | 0.930 | 0.552 | 0.988 | 0.929 |
| T2 | 1.432 | 0.989 | 1.044 | 1.160 | 1.803 | 0.865 | 1.213 | 1.274 |
| T3 | 2.032 | 2.037 | 2.190 | 1.929 | 2.336 | 2.077 | 1.895 | 1.899 |
| T4 | 2.474 | 2.478 | 2.417 | 2.364 | 3.037 | 2.552 | 2.351 | 2.268 |
| | Relative energies of excited states TDA/TDDFT | | | | | | | |
| S1 | 2.226 | 2.022 | 2.047 | 2.106 | 1.973 | 1.979 | 2.134 | 2.161 |
| S2 | 2.626 | 2.496 | 2.405 | 2.550 | 2.497 | 2.472 | 2.567 | 2.557 |
| S3 | 3.454 | 3.414 | 3.450 | 3.350 | 3.378 | 3.445 | 3.364 | 3.330 |
| T1 | 1.538 | 1.343 | 1.339 | 1.494 | 1.007 | 1.253 | 1.534 | 1.587 |
| T2 | 1.763 | 1.546 | 1.374 | 1.700 | 1.715 | 1.498 | 1.779 | 1.725 |
| T3 | 2.191 | 2.161 | 2.354 | 2.021 | 2.223 | 2.208 | 1.959 | 1.986 |
| T4 | 2.612 | 2.605 | 2.584 | 2.477 | 2.728 | 2.679 | 2.458 | 2.375 |

**Table S2** Relative energies calculated at the stationary point for the basal state and each excited state for ZnTMEe6. The energies were calculated using CAM-B3LYP/def2-SVP.

| | @S0 | @S1 | @S2 | @S3 | @T1 | @T2 | @T3 | @T4 |
|---|---|---|---|---|---|---|---|---|
| State | Relative energies of excited states full TDDFT | | | | | | | |
| S1 | 2.075 | 1.825 | 1.867 | 2.003 | 1.730 | 1.836 | 2.062 | 2.098 |
| S2 | 2.556 | 2.362 | 2.202 | 2.463 | 2.384 | 2.133 | 2.529 | 2.483 |
| S3 | 3.174 | 3.145 | 3.131 | 3.037 | 3.132 | 3.031 | 3.048 | 3.033 |
| T1 | 1.062 | 0.651 | 0.748 | 0.987 | 0.014 | 0.675 | 1.042 | 1.127 |
| T2 | 1.604 | 1.241 | 1.029 | 1.460 | 1.459 | 0.858 | 1.592 | 1.509 |
| T3 | 2.107 | 2.126 | 2.157 | 1.950 | 2.190 | 2.105 | 1.901 | 1.929 |
| T4 | 2.511 | 2.342 | 2.285 | 2.444 | 2.427 | 2.342 | 2.445 | 2.321 |
| | Relative energies of excited states TDA/TDDFT | | | | | | | |
| S1 | 2.235 | 2.017 | 2.049 | 2.169 | 1.931 | 2.022 | 2.221 | 2.249 |
| S2 | 2.680 | 2.511 | 2.369 | 2.588 | 2.526 | 2.317 | 2.645 | 2.603 |
| S3 | 3.443 | 3.330 | 3.217 | 3.327 | 3.241 | 3.114 | 3.345 | 3.320 |
| T1 | 1.486 | 1.197 | 1.262 | 1.440 | 1.021 | 1.235 | 1.497 | 1.566 |
| T2 | 1.919 | 1.703 | 1.486 | 1.878 | 1.764 | 1.358 | 1.962 | 1.900 |
| T3 | 2.295 | 2.283 | 2.379 | 2.105 | 2.288 | 2.343 | 2.078 | 2.099 |
| T4 | 2.730 | 2.578 | 2.483 | 2.584 | 2.509 | 2.515 | 2.578 | 2.451 |

**Table S3** Relative energies calculated at the stationary point for the basal state and each excited state for PyrTMEe6. The energies were calculated using CAM-B3LYP/def2-SVP.

| | **@S0** | **@S1** | **@S2** | **@S3** | **@S4** | **@T1** | **@T2** | **@T3** | **@T4** |
|---|---|---|---|---|---|---|---|---|---|
| State | Relative energies of excited states full TDDFT | | | | | | | | |
| S1 | 1.921 | 1.812 | 1.818 | 1.925 | 1.909 | 1.745 | 1.758 | 1.933 | 1.954 |
| S2 | 2.408 | 2.320 | 2.190 | 2.425 | 2.408 | 2.263 | 2.286 | 2.419 | 2.398 |
| S3 | 3.027 | 3.042 | 3.151 | 2.425 | 2.963 | 2.999 | 3.034 | 2.989 | 2.993 |
| S4 | 3.105 | 3.122 | 3.237 | 3.075 | 3.034 | 3.093 | 3.139 | 3.054 | 3.085 |
| T1 | 0.831 | 0.569 | 0.191 | 0.875 | 0.872 | 0.876 | 0.436 | 0.939 | 0.841 |
| T2 | 1.141 | 0.964 | 1.026 | 1.138 | 1.122 | 1.702 | 0.840 | 1.171 | 1.203 |
| T3 | 2.026 | 2.054 | 2.248 | 1.984 | 1.942 | 2.337 | 2.095 | 1.889 | 1.918 |
| T4 | 2.446 | 2.489 | 2.502 | 2.404 | 2.386 | 3.000 | 2.558 | 2.358 | 2.284 |
| | Relative energies of excited states TDA/TDDFT | | | | | | | | |
| S1 | 2.103 | 2.009 | 2.015 | 2.109 | 2.090 | 1.960 | 1.966 | 2.109 | 2.127 |
| S2 | 2.557 | 2.483 | 2.385 | 2.430 | 2.549 | 2.429 | 2.456 | 2.555 | 2.539 |
| S3 | 3.046 | 3.073 | 3.203 | 2.566 | 2.972 | 3.019 | 3.053 | 3.031 | 3.084 |
| S4 | 3.410 | 3.413 | 3.437 | 3.188 | 3.357 | 3.385 | 3.443 | 3.349 | 3.317 |
| T1 | 1.448 | 1.316 | 1.288 | 1.429 | 1.458 | 0.997 | 1.232 | 1.499 | 1.531 |
| T2 | 1.634 | 1.511 | 1.329 | 1.727 | 1.682 | 1.642 | 1.458 | 1.756 | 1.659 |
| T3 | 2.149 | 2.182 | 2.354 | 2.067 | 2.036 | 2.224 | 2.229 | 1.953 | 2.024 |
| T4 | 2.559 | 2.608 | 2.572 | 2.414 | 2.495 | 2.788 | 2.680 | 2.464 | 2.386 |

## C Energy diagrams

In this section, additional energy diagrams (Figure S1-Figure S3) are presented. They show the relative energies of excited states with the Tamm-Dancoff approximation at each stationary point from Tables S1-S3. The energy of the optimized state is presented in bold.

**Figure S1** Energy diagram for adiabatic energies of each stationary point for TMEe6. The energies were calculated using TDA/CAM-B3LYP/def2-SVP.

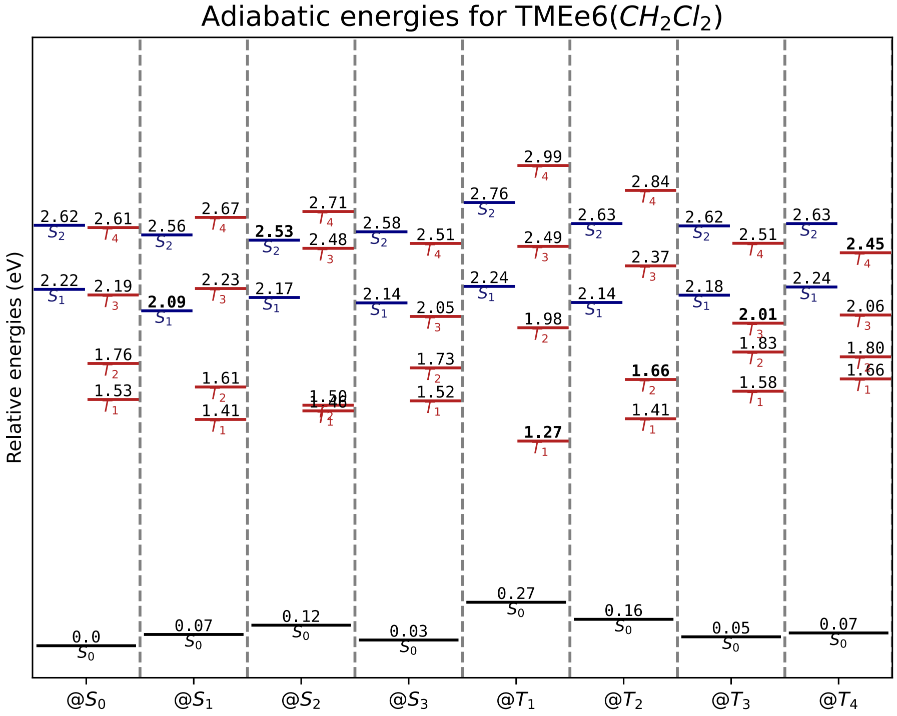


**Figure S2** Energy diagram for adiabatic energies of each stationary point for ZnTMEe6. The energies were calculated using TDA/CAM-B3LYP/def2-SVP.

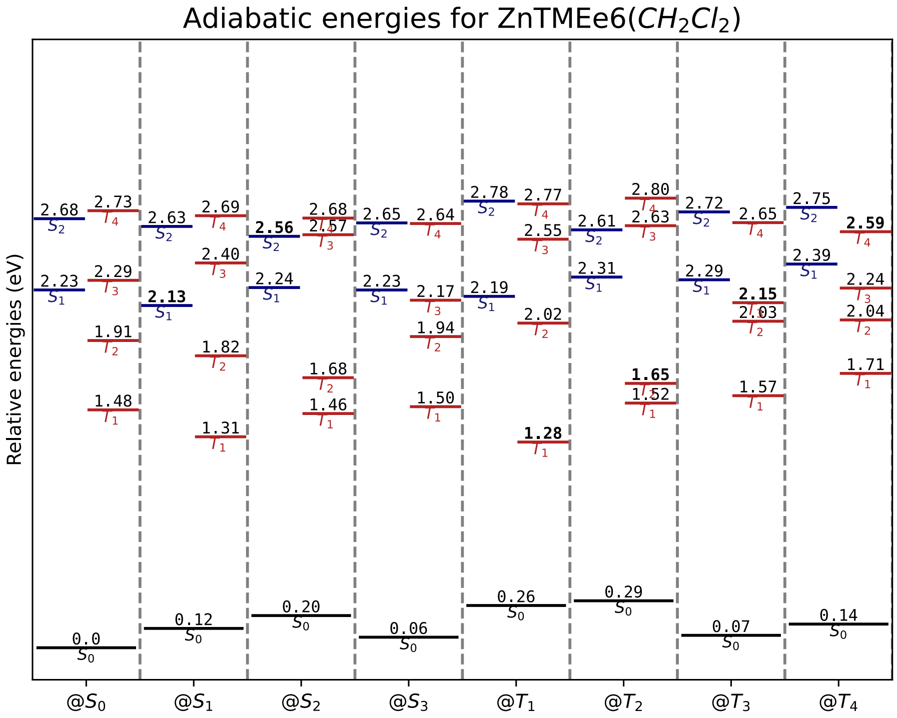

**Figure S3** Energy diagram for adiabatic energies of each stationary point for PyrTMEe6. The energies were calculated using TDA/CAM-B3LYP/def2-SVP.

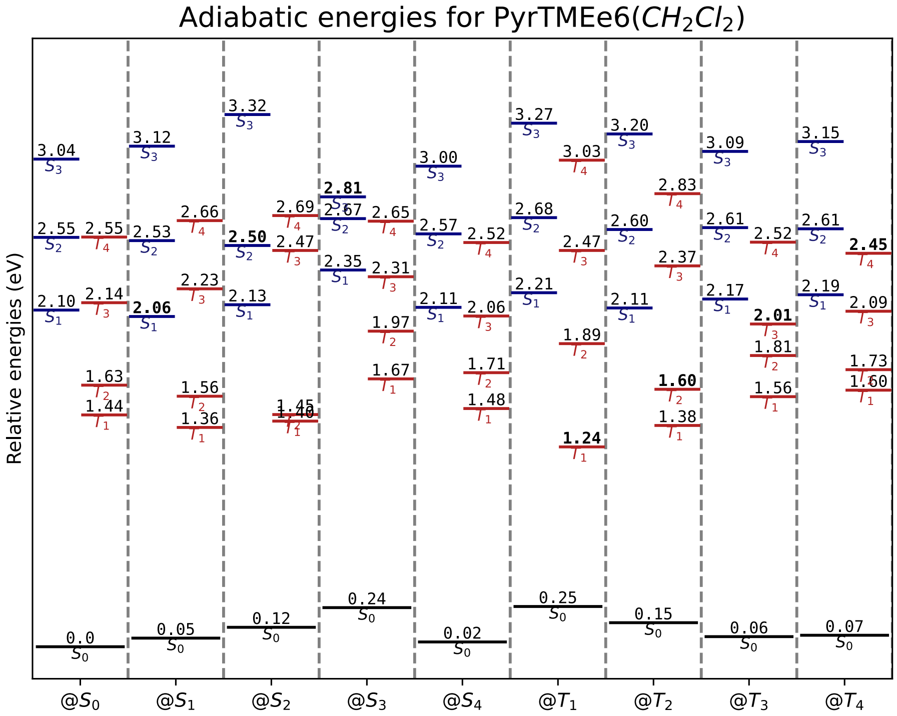

# D Calculated spectra with vibronic progression

In this section, the calculated spectra for absorption, fluorescence and phosphorescence of the molecules are shown together with the vibronic progression calculated with an inhomogeneous band width of 150 $cm^{-1}$ (298K) and 1 $cm^{-1}$ (0K). Computed spectra are compared with experimental data if available.

## D.1 Absorption spectra

**Figure S4** Calculated absorption spectrum of TMEe6 for $S_1$ transitions. Adiabatic and vertical energies calculated with CAM-B3LYP/def2-SVP are shown as vertical lines. The experimental spectrum is shown as dotted lines, taken from Blanco Ramírez and Calderón Hernández [1].

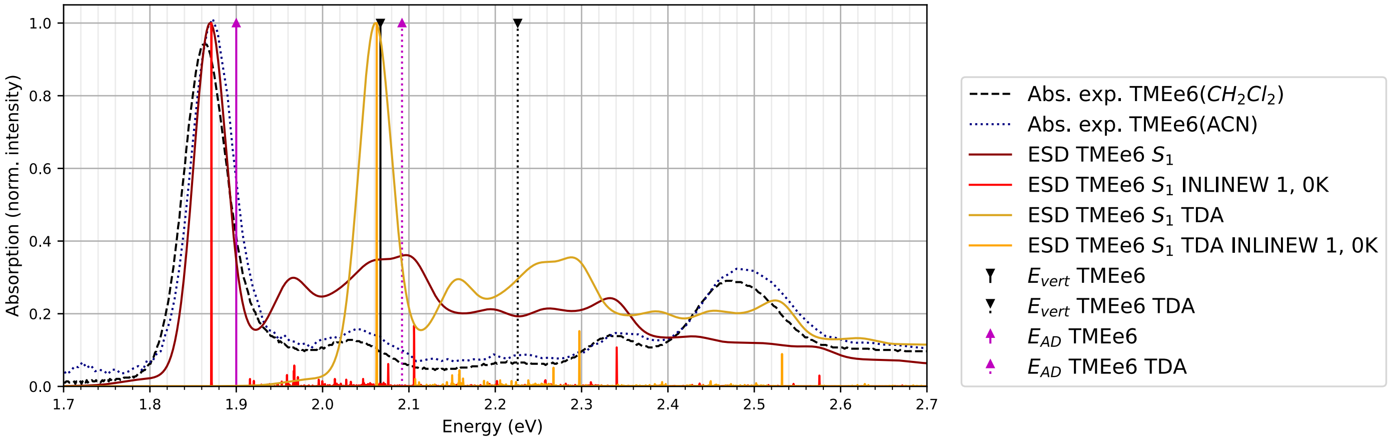


**Figure S5** Calculated absorption spectrum of ZnTMEe6 for $S_1$ transitions. Adiabatic and vertical energies calculated with CAM-B3LYP/def2-SVP are shown as vertical lines. The experimental spectrum is shown as dotted lines, taken from Muñoz [2].

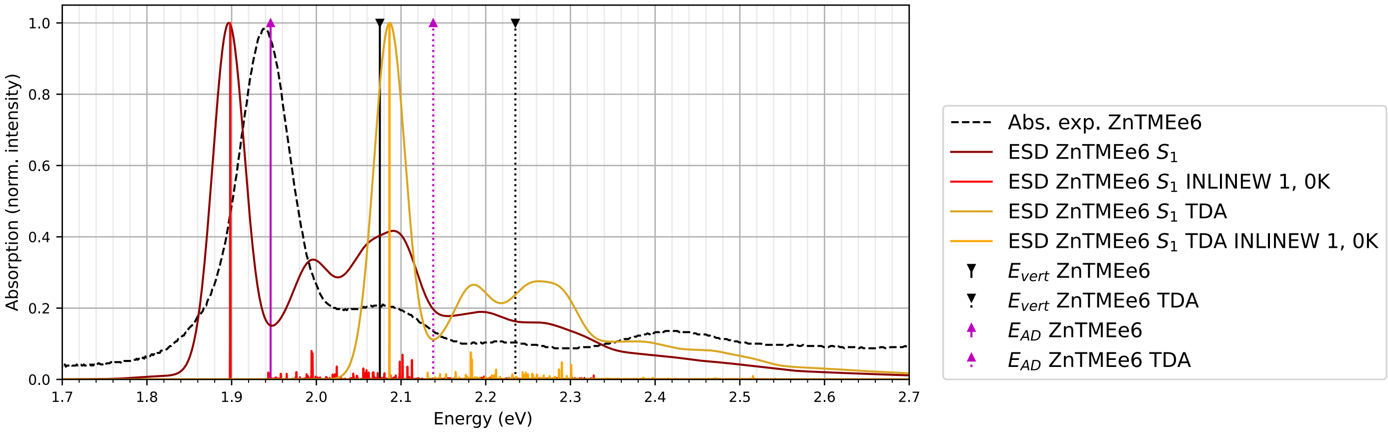

**Figure S6** Calculated absorption spectrum of PyrTMEe6 for $S_1$ transitions. Adiabatic and vertical energies calculated with CAM-B3LYP/def2-SVP are shown as vertical lines. The experimental spectrum is shown as dotted lines, taken from Tarazona Cáceres [3].

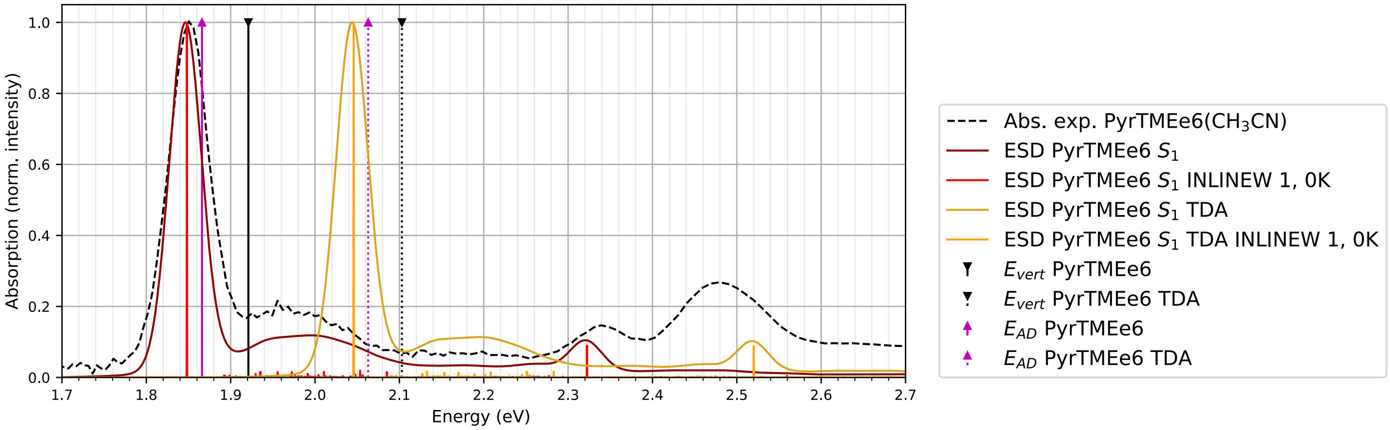


**Figure S7** Calculated absorption spectrum of TMEe6 for $S_2$ transitions. Adiabatic and vertical energies calculated with CAM-B3LYP/def2-SVP are shown as vertical lines. The experimental spectrum is shown as dotted lines, taken from Blanco Ramírez and Calderón Hernández [1].

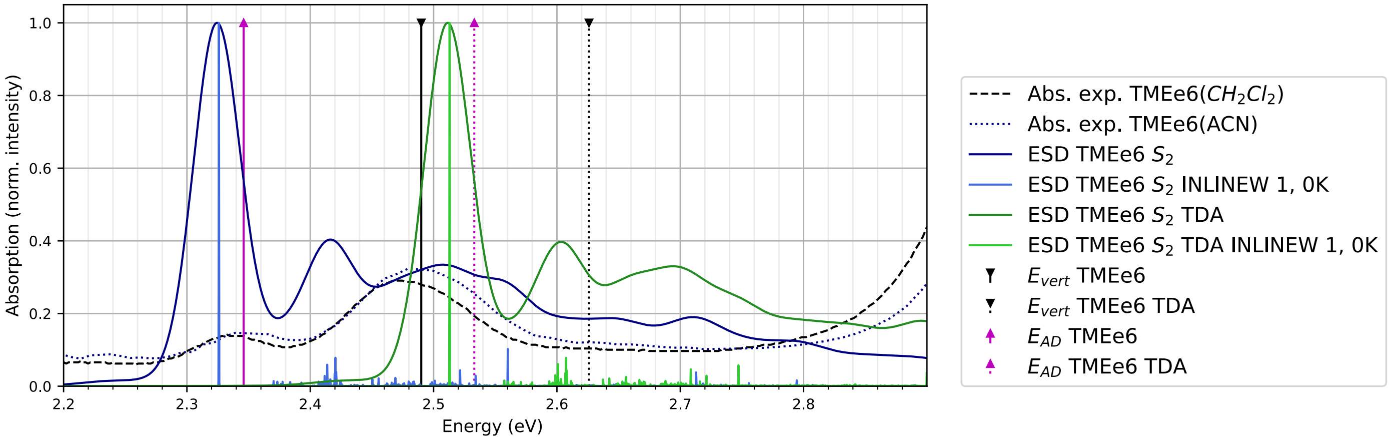


**Figure S8** Calculated absorption spectrum of ZnTMEe6 for $S_2$ transitions. Adiabatic and vertical energies calculated with CAM-B3LYP/def2-SVP are shown as vertical lines. The experimental spectrum is shown as dotted lines, taken from Muñoz [2].

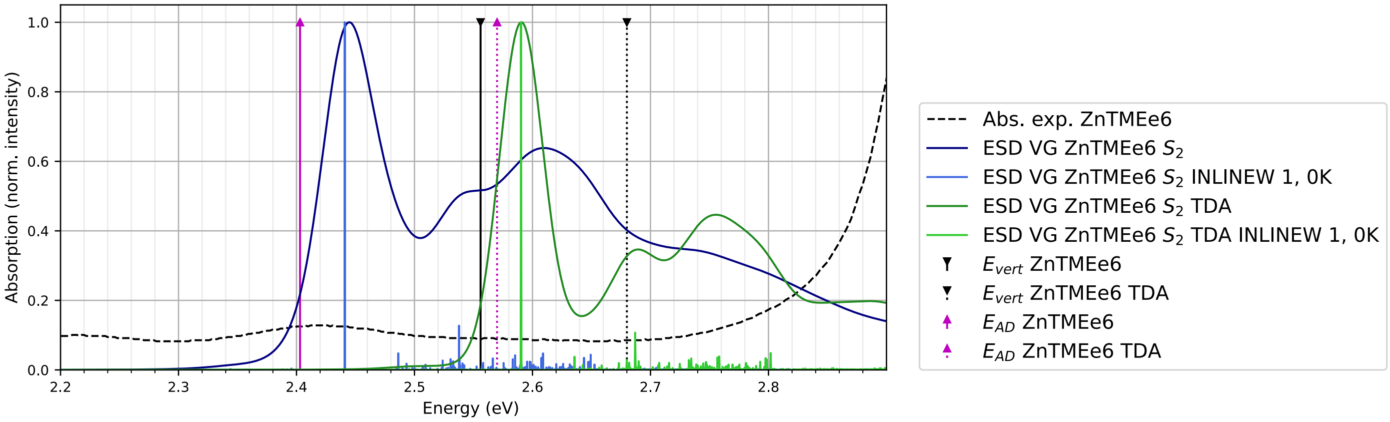

**Figure S9** Calculated absorption spectrum of PyrTMEe6 for $S_2$ transitions. The experimental spectrum is shown as dotted lines. Adiabatic and vertical energies calculated with CAM-B3LYP/def2-SVP are shown as vertical lines. Experimental spectrum taken from Tarazona Cáceres [3].

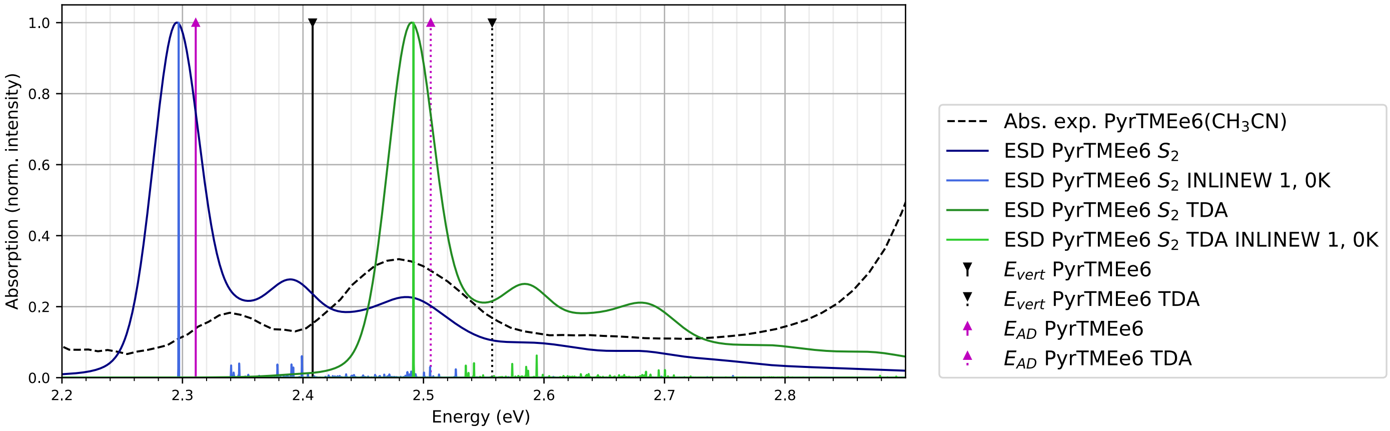


### D.1.1 Herzberg-Teller contributions to the calculated absorption spectra

In this section, we present the calculated absorption spectra, including Herzberg-Teller effects (FCHT) and with only Franck-Condon (FC) contributions.

**Table S4** Herzberg-Teller contributions to the calculated absorption spectra.

| Transition | $S_0 \rightarrow S_1$ | $S_0 \rightarrow S_2$ |
|---|---|---|
| TMEe6 | 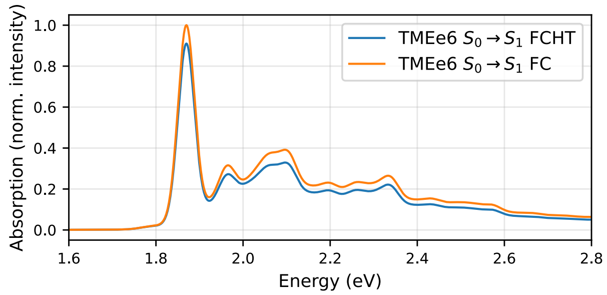 | 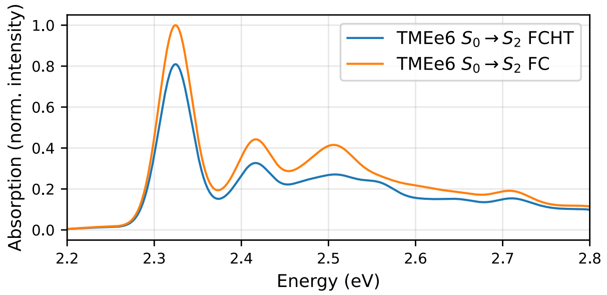 |
| ZnTMEe6 | 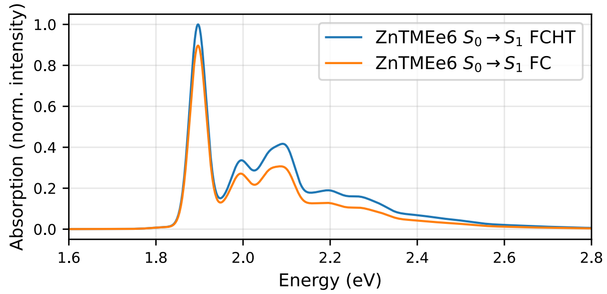 | 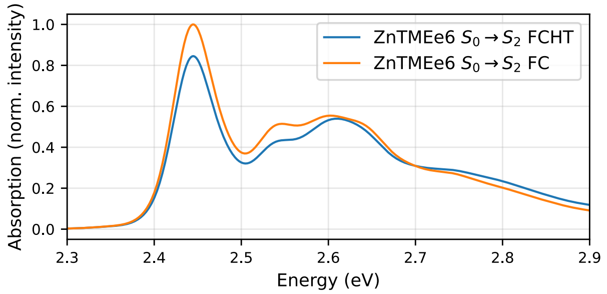 |
| PyrTMEe6 | 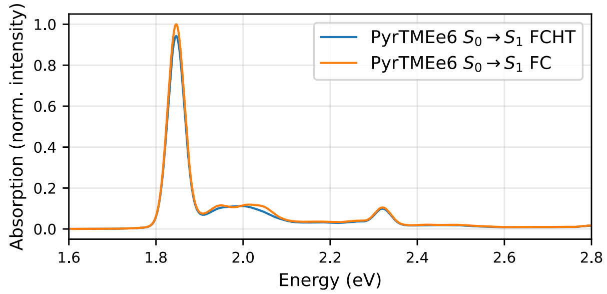 | 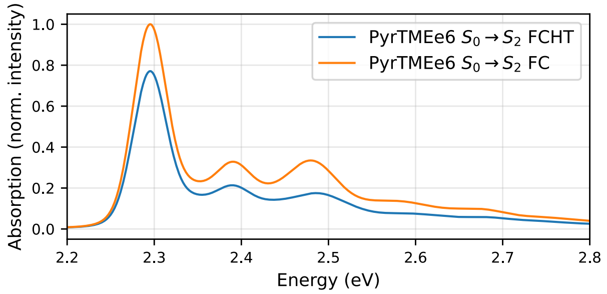 |

## D.2 Fluorescence spectra

**Figure S10** Calculated fluorescence spectrum of TMEe6. Adiabatic and vertical energies calculated with CAM-B3LYP/def2-SVP are shown as vertical lines.

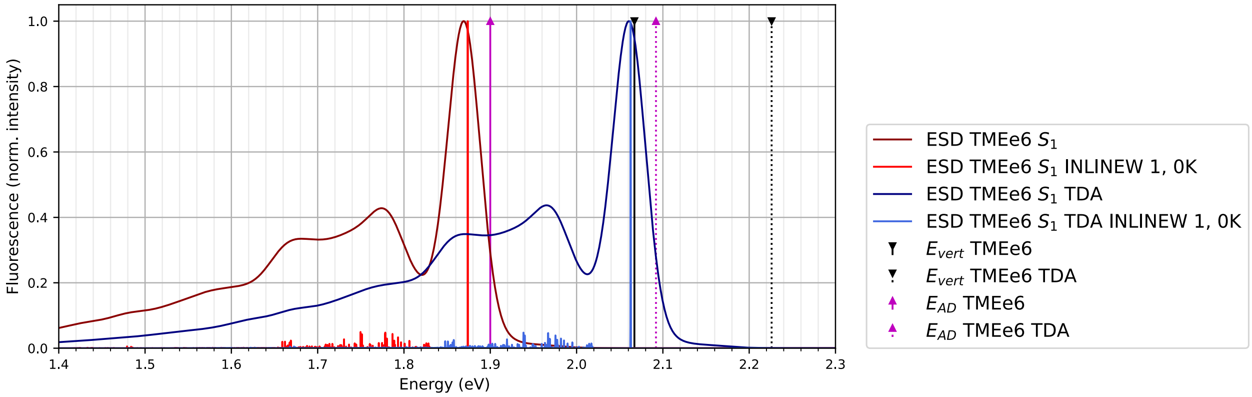


**Figure S11** Calculated fluorescence spectrum of ZnTMEe6. Adiabatic and vertical energies calculated with CAM-B3LYP/def2-SVP are shown as vertical lines.

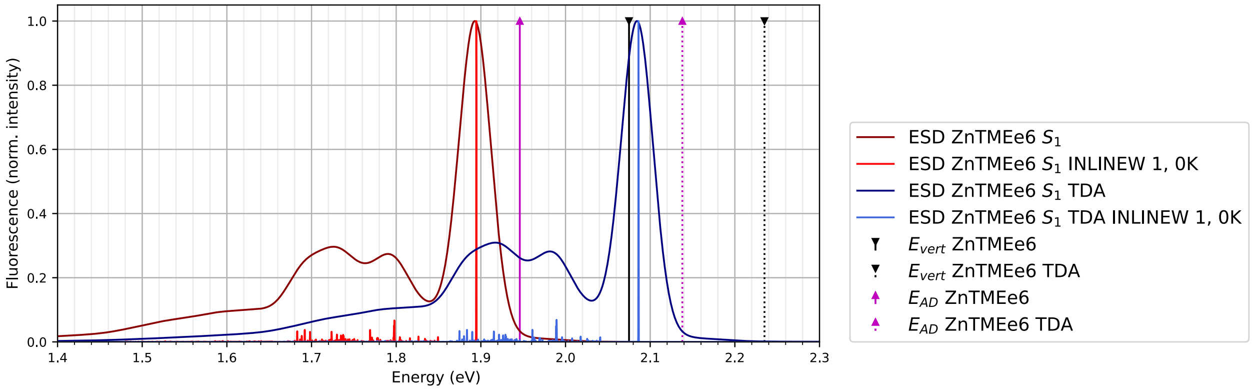


**Figure S12** Calculated fluorescence spectrum of PyrTMEe6. Adiabatic and vertical energies calculated with CAM-B3LYP/def2-SVP are shown as vertical lines.

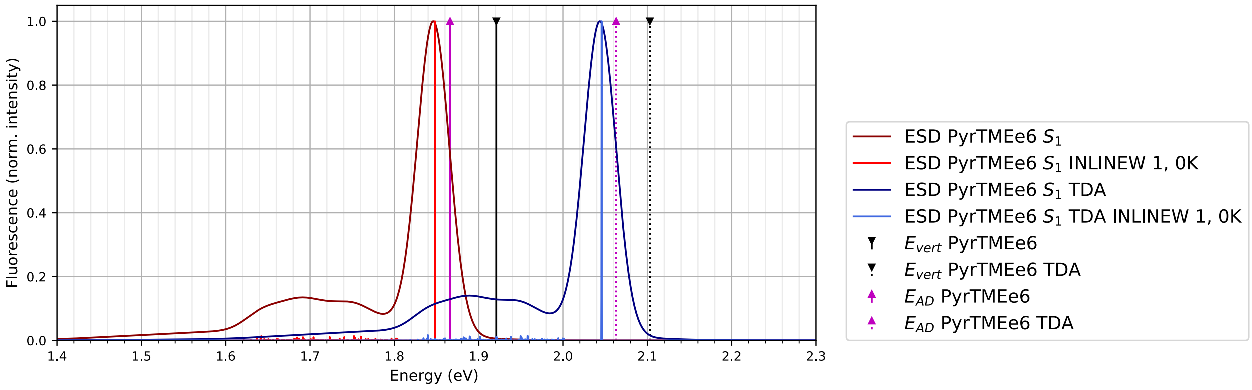

## D.3 Phosphorescence spectra

**Figure S13** Calculated phosphorescence spectrum of TMEe6, normalized over the mean of the spectra of each sublevel.

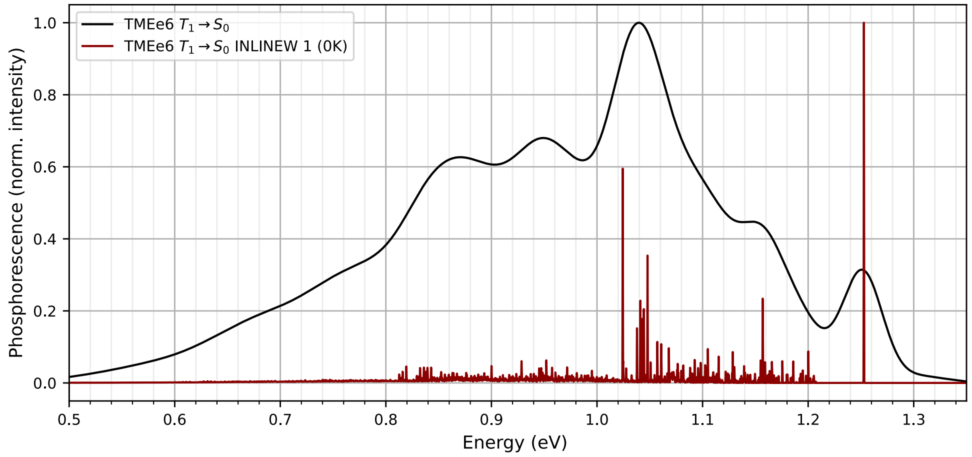


**Figure S14** Calculated phosphorescence spectrum of ZnTMEe6, normalized over the mean of the spectra of each sublevel.

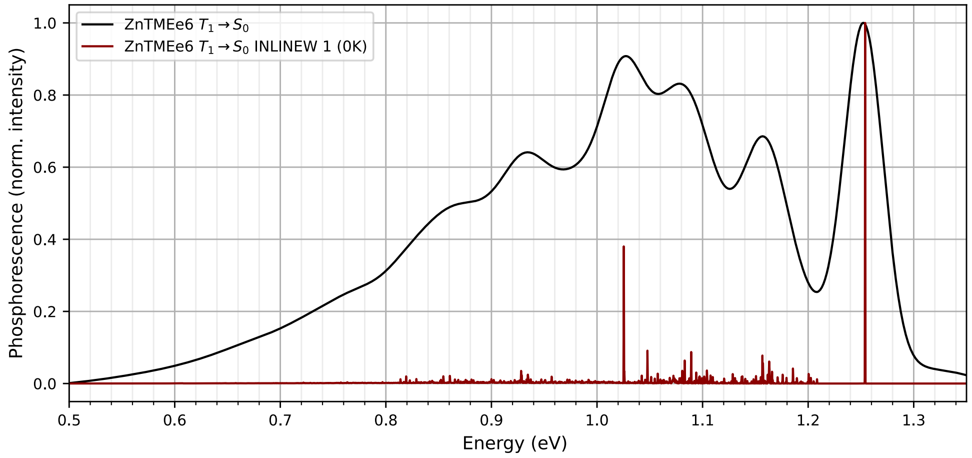


**Figure S15** Calculated phosphorescence spectrum of PyrTMEe6, normalized over the mean of the spectra of each sublevel.

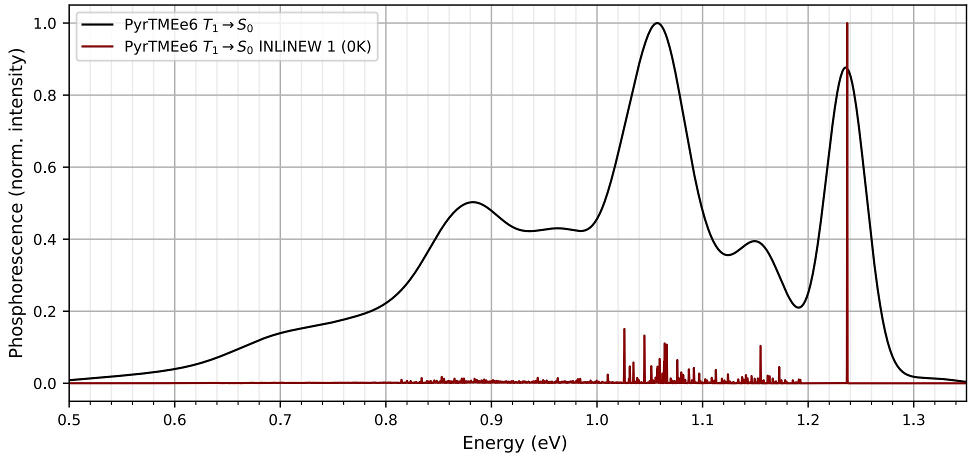

## D.4 Effect of the selection of conformer

In this section, the effect of the selected conformer on the absorption and fluorescence spectra of TMEe6 is investigated. The geometries of the conformers are shown in Figure S16.

**Figure S16** Geometries of the chosen conformers to test their effect on the absorption spectra of TMEe6 and their respective Boltzmann weights (%W).

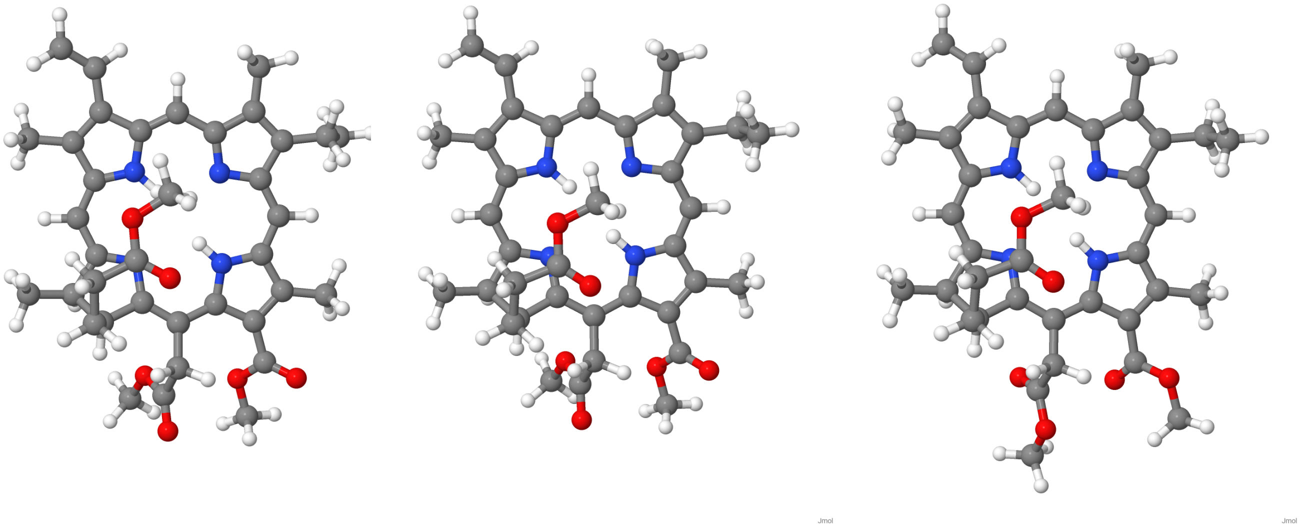

**(a)** CONF 84, %W 17.11 (Minimum energy conformer)  **(b)** CONF 78, %W 16.95  **(c)** CONF 11, %W 13.29

Each of the above conformers was optimized using CAM-B3LYP/def2-SVP, using DFT for their ground state $S_0$, and TD-DFT for their first excited singlet state $S_1$. Their absorption spectra for the $Q_y$ bands ($S_1$) and $Q_x$ bands ($S_2$) are shown in Figure S17. Fluorescence spectra are shown in Figure S18.

**Figure S17** Calculated absorption spectra for the $Q_y$ band, $S_0 \rightarrow S_1$ and the $Q_x$ band, $S_0 \rightarrow S_2$.

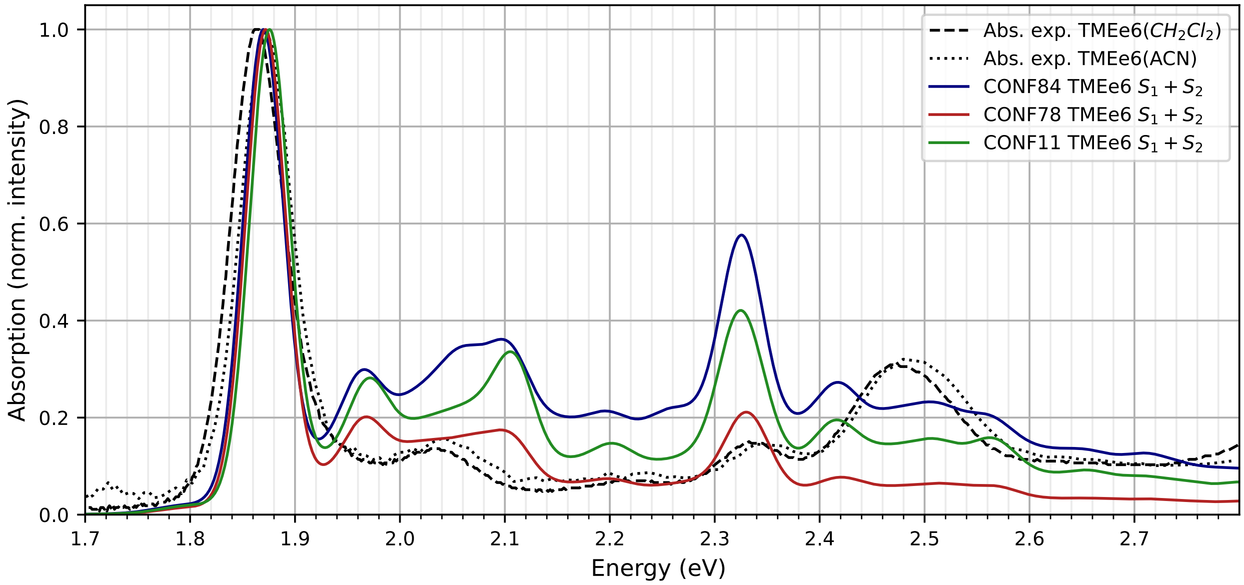

**Figure S18** Calculated fluorescence spectra of the chosen conformers.

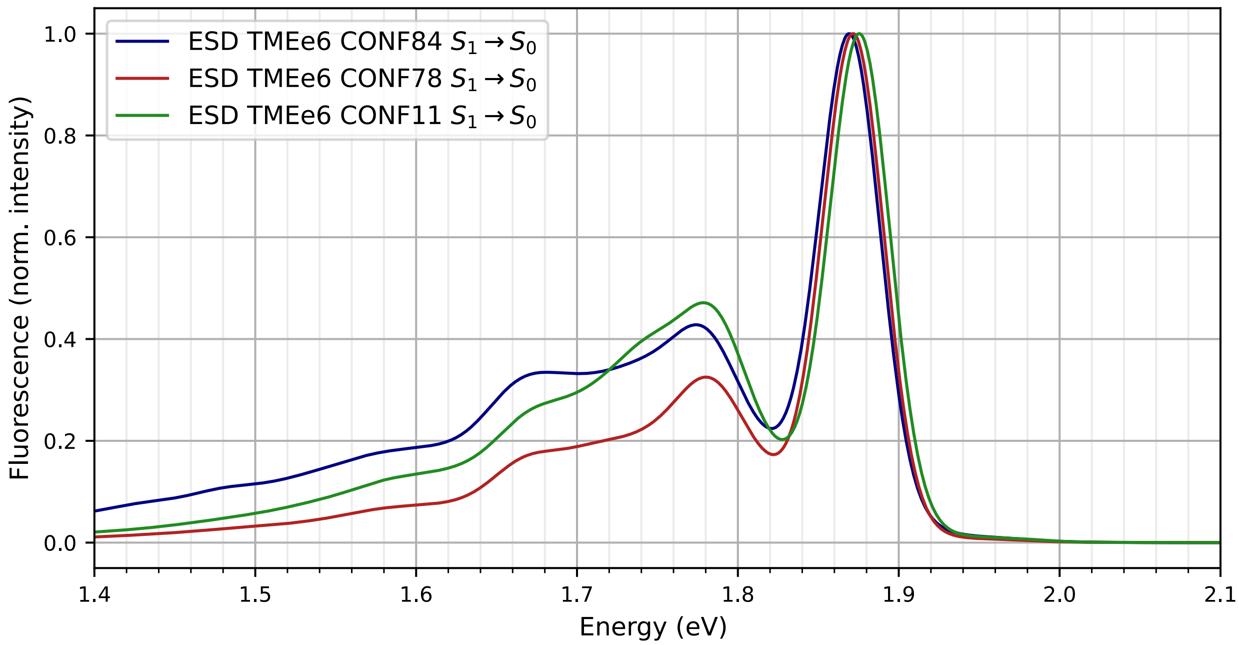


## E Calculated transition rate constants

In the main body of the article, brief tables are presented with the rates for the studied processes. This section displays tables with more details on phosphorescence and ISC rates, including sublevels for each channel.

### E.1 Phosphorescence rate constants

**Table S5** Phosphorescence rate constants with sublevel detail.

| **Molecule** | TMEe6 | | ZnTMEe6 | | PyrTMEe6 | |
|---|---|---|---|---|---|---|
| **Channel** | $T_1 \rightarrow S_0$ | | $T_1 \rightarrow S_0$ | | $T_1 \rightarrow S_0$ | |
| **Sublevel** | $k_P$ ($s^{-1}$) | HT cont. (%) | $k_P$ ($s^{-1}$) | HT cont. (%) | $k_P$ ($s^{-1}$) | HT cont. (%) |
| 1 | 1.46 | 90.38 | 4.09 | 98.74 | 0.97 | 89.50 |
| 0 | 0.29 | 49.55 | 4.52 | 93.13 | 1.37 | 98.85 |
| -1 | 3.49 | 93.68 | 15.64 | 37.04 | 24.39 | 89.89 |
| Average | 1.75 | 90.33[a] | 8.09 | 57.90[a] | 8.91 | 90.34[a] |

[a] As the weighted arithmetic mean of the Herzberg-Teller contribution (%).

### E.2 Intersystem crossing rate constants

The following tables contain details related to ISC rate calculations. SOCME derivatives for each vibrational mode are provided in the files named ISC_TMEe6_dSOCME.xls, ISC_ZnTMEe6_dSOCME.xls, ISC_PyrTMEe6_dSOCME.xls.

**Table S6** Intersystem crossing rate constants for TMEe6 with sublevel detail.

| Channel | $S_1 \rightsquigarrow T_1$ | | | $S_1 \rightsquigarrow T_2$ | | | $S_1 \rightsquigarrow T_3$ | | |
|---|---|---|---|---|---|---|---|---|---|
| Sublevel | $k_{ISC}$ ($s^{-1}$) | SOCME | HT cont. (%) | $k_{ISC}$ ($s^{-1}$) | SOCME | HT cont. (%) | $k_{ISC}$ ($s^{-1}$) | SOCME | HT cont. (%) |
| 1 | $5.44\times10^6$ | 0.173 | 64.81 | $5.39\times10^7$ | 0.504 | 37.88 | $2.11\times10^6$ | 0.090 | 80.95 |
| 0 | $1.38\times10^6$ | 0.094 | 59.36 | $4.78\times10^6$ | 0.138 | 47.35 | $1.81\times10^4$ | 0.011 | 63.48 |
| -1 | $5.40\times10^6$ | 0.173 | 64.54 | $5.40\times10^7$ | 0.504 | 38.01 | $2.12\times10^6$ | 0.090 | 81.06 |
| Total | $1.22\times10^7$ | - | $64.07^a$ | $1.13\times10^8$ | - | $38.34^a$ | $4.25\times10^6$ | - | $80.93^a$ |

[a]As the weighted arithmetic mean of the Herzberg-Teller contribution (%).

**Table S7** Intersystem crossing rate constants for ZnTMEe6 with sublevel detail.

| Channel | $S_1 \rightsquigarrow T_1$ | | | $S_1 \rightsquigarrow T_2$ | | | $S_1 \rightsquigarrow T_3$ | | |
|---|---|---|---|---|---|---|---|---|---|
| Sublevel | $k_{ISC}$ ($s^{-1}$) | SOCME | HT cont. (%) | $k_{ISC}$ ($s^{-1}$) | SOCME | HT cont. (%) | $k_{ISC}$ ($s^{-1}$) | SOCME | HT cont. (%) |
| 1 | $2.11\times10^6$ | 0.096 | 46.93 | $4.38\times10^6$ | 0.254 | 77.67 | $1.18\times10^8$ | 0.287 | 99.52 |
| 0 | $3.23\times10^6$ | 0.125 | 41.80 | $1.27\times10^7$ | 0.604 | 56.47 | $1.64\times10^7$ | 1.010 | 57.74 |
| -1 | $2.11\times10^6$ | 0.096 | 46.85 | $4.37\times10^6$ | 0.254 | 77.64 | $1.19\times10^8$ | 0.287 | 99.53 |
| Total | $7.46\times10^6$ | - | $44.68^a$ | $2.14\times10^7$ | - | $65.11^a$ | $2.54\times10^8$ | - | $96.83^a$ |

[a]As the weighted arithmetic mean of the Herzberg-Teller contribution (%)

**Table S8** Intersystem crossing rate constants for PyrTMEe6 with sublevel detail.

| Channel | $S_1 \rightsquigarrow T_1$ | | | $S_1 \rightsquigarrow T_2$ | | | $S_1 \rightsquigarrow T_3$ | | |
|---|---|---|---|---|---|---|---|---|---|
| Sublevel | $k_{ISC}$ ($s^{-1}$) | SOCME | HT cont. (%) | $k_{ISC}$ ($s^{-1}$) | SOCME | HT cont. (%) | $k_{ISC}$ ($s^{-1}$) | SOCME | HT cont. (%) |
| 1 | $3.21\times10^7$ | 0.195 | 76.56 | $4.79\times10^7$ | 0.521 | 52.99 | $9.09\times10^6$ | 0.093 | 66.41 |
| 0 | $9.63\times10^5$ | 0.035 | 74.79 | $3.46\times10^6$ | 0.105 | 73.50 | $1.81\times10^5$ | 0.003 | 98.59 |
| -1 | $3.16\times10^7$ | 0.195 | 76.20 | $4.79\times10^7$ | 0.521 | 53.06 | $9.16\times10^6$ | 0.093 | 66.64 |
| Total | $6.46\times10^7$ | - | $76.36^a$ | $9.93\times10^7$ | - | $53.74^a$ | $1.84\times10^7$ | - | $66.84^a$ |

[a]As the weighted arithmetic mean of the Herzberg-Teller contribution (%)

### E.2.1 Effect of the energy gap ΔE(S-T) on the intersystem crossing rate constants $k_{ISC}$

To investigate the effect of the change of the energy gap between singlet and triplet states ΔE(S-T) on $k_{ISC}$, extra calculations were performed, changing the DELE parameter (adiabatic energy difference between singlet and triplet states) by ±0.1 eV. The results are shown in the following tables and figures.

**Table S9** Intersystem crossing rate constants of TMEe6, calculated with various singlet-triplet energy differences ΔE(S-T).

| Transition | ΔE(S-T) (eV) | $k_{ISC}$ for each sublevel ($s^{-1}$) 1 | 0 | -1 | Total $k_{ISC}{}^a$ ($s^{-1}$) |
|---|---|---|---|---|---|
| $S_1 \rightsquigarrow T_1$ | 0.714 | $3.13\times10^6$ | $8.47\times10^6$ | $3.10\times10^6$ | $1.47\times10^7$ |
| | 0.814 | $5.44\times10^6$ | $1.39\times10^6$ | $5.40\times10^6$ | $1.22\times10^7$ |
| | 0.914 | $4.30\times10^6$ | $1.16\times10^6$ | $4.26\times10^6$ | $9.72\times10^6$ |
| $S_1 \rightsquigarrow T_2$ | 0.329 | $5.08\times10^7$ | $4.56\times10^6$ | $5.08\times10^7$ | $1.06\times10^8$ |
| | 0.429 | $5.39\times10^7$ | $4.78\times10^6$ | $5.40\times10^7$ | $1.13\times10^8$ |
| | 0.529 | $5.04\times10^7$ | $4.60\times10^6$ | $5.05\times10^7$ | $1.06\times10^8$ |
| $S_1 \rightsquigarrow T_3$ | -0.022 | $1.50\times10^5$ | $1.56\times10^3$ | $1.51\times10^5$ | $3.03\times10^5$ |
| | 0.078 | $2.11\times10^6$ | $1.81\times10^4$ | $2.12\times10^6$ | $4.25\times10^6$ |
| | 0.178 | $4.11\times10^6$ | $4.24\times10^4$ | $4.14\times10^6$ | $8.29\times10^6$ |

[a] Calculated as the sum of the $k_{ISC}$ for each sublevel.

**Figure S19** Intersystem crossing rate constants for TMEe6, calculated with various singlet-triplet energy differences ΔE(S-T), for each one of the $S_1 \rightsquigarrow \{T_1, T_2, T_3\}$ transitions. The bar chart to the right employs a logarithmic scale.

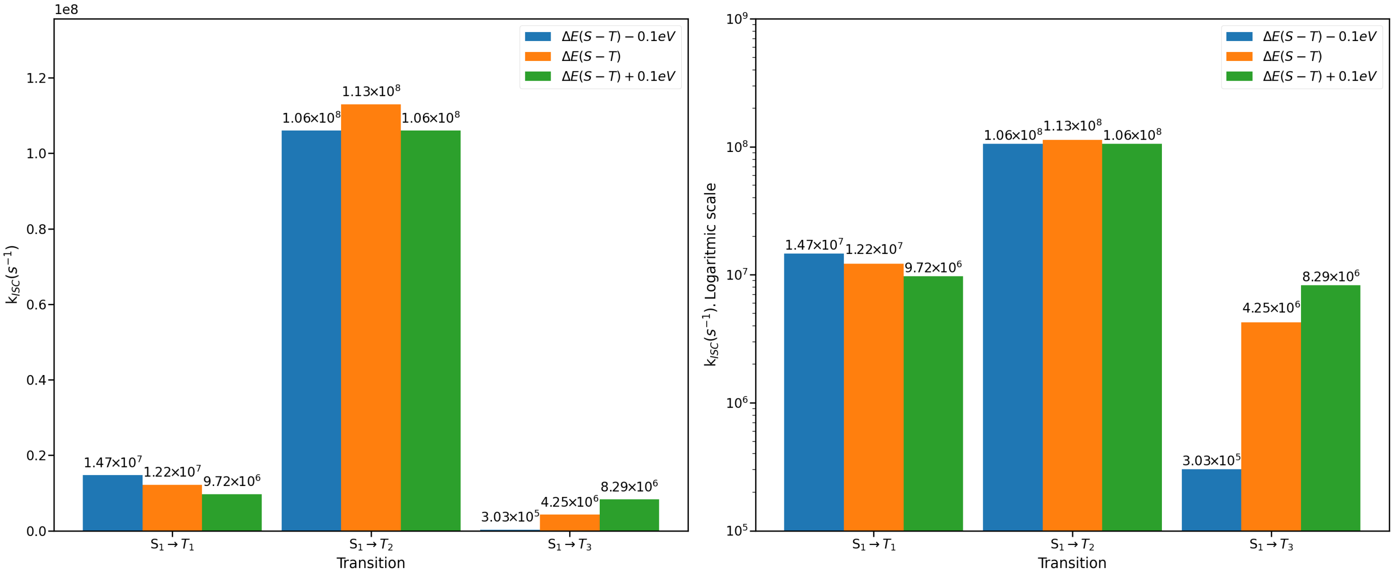


**Table S10** Intersystem crossing rate constants of ZnTMEe6, calculated with various singlet-triplet energy differences ΔE(S-T).

| Transition | ΔE(S-T) (eV) | $k_{ISC}$ for each sublevel ($s^{-1}$) 1 | 0 | -1 | Total $k_{ISC}$[a] ($s^{-1}$) |
|---|---|---|---|---|---|
| $S_1 \rightsquigarrow T_1$ | 0.753 | $1.76\times10^6$ | $2.64\times10^6$ | $1.76\times10^6$ | $6.16\times10^6$ |
| | 0.853 | $2.11\times10^6$ | $3.23\times10^6$ | $2.11\times10^6$ | $7.45\times10^6$ |
| | 0.953 | $2.16\times10^6$ | $3.24\times10^6$ | $2.16\times10^6$ | $7.56\times10^6$ |
| $S_1 \rightsquigarrow T_2$ | 0.386 | $2.17\times10^6$ | $6.09\times10^6$ | $2.17\times10^6$ | $1.04\times10^7$ |
| | 0.486 | $4.38\times10^6$ | $1.27\times10^7$ | $4.37\times10^6$ | $2.15\times10^7$ |
| | 0.586 | $6.35\times10^6$ | $1.72\times10^7$ | $6.34\times10^6$ | $2.99\times10^7$ |
| $S_1 \rightsquigarrow T_3$ | -0.118 | $1.20\times10^8$ | $1.63\times10^7$ | $1.21\times10^8$ | $2.57\times10^8$ |
| | -0.018 | $1.18\times10^8$ | $1.64\times10^7$ | $1.19\times10^8$ | $2.53\times10^8$ |
| | 0.082 | $1.22\times10^8$ | $5.55\times10^7$ | $1.23\times10^8$ | $3.01\times10^8$ |

[a] Calculated as the sum of the $k_{ISC}$ for each sublevel.

**Figure S20** Intersystem crossing rate constants of ZnTMEe6, calculated with various singlet-triplet energy differences ΔE(S-T), for each of the $S_1 \rightsquigarrow \{T_1, T_2, T_3\}$ transitions. The bar chart to the right employs a logarithmic scale.

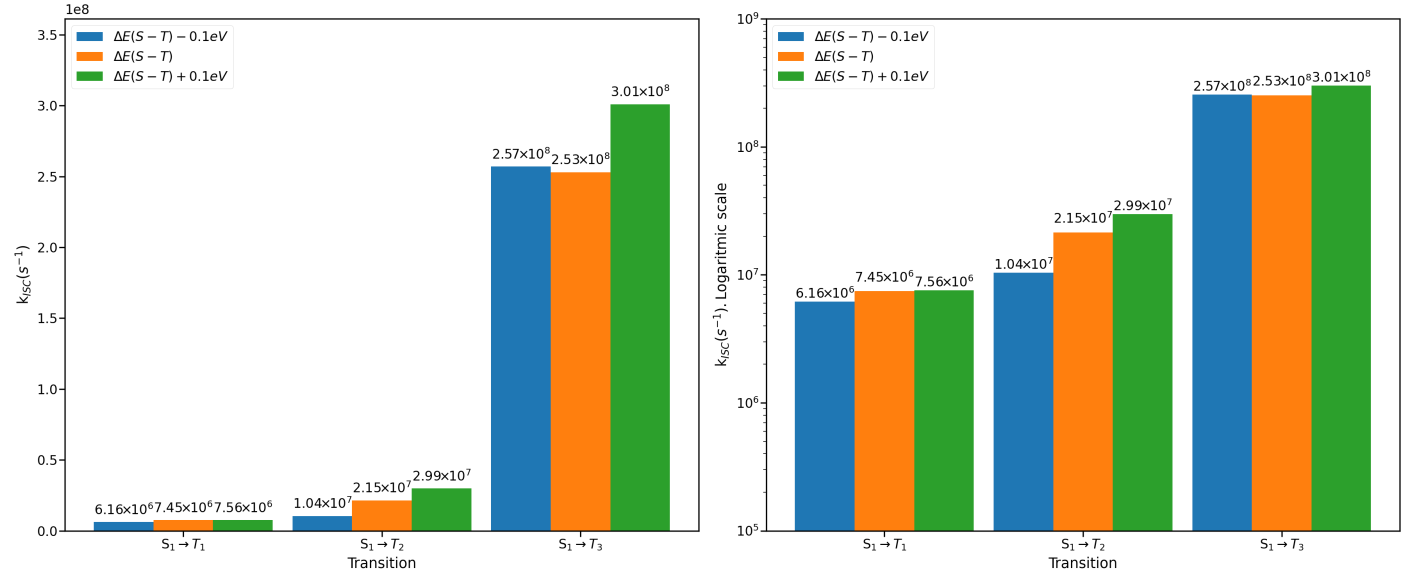


**Table S11** Intersystem crossing rate constants of PyrTMEe6, calculated with various singlet-triplet energy differences ΔE(S-T).

| Transition | ΔE(S-T) (eV) | $k_{ISC}$ for each sublevel ($s^{-1}$) | | | Total $k_{ISC}$[a] ($s^{-1}$) |
|---|---|---|---|---|---|
| | | 1 | 0 | -1 | |
| $S_1 \rightsquigarrow T_1$ | 0.714 | $3.62\times10^{7}$ | $1.10\times10^{6}$ | $3.56\times10^{7}$ | $7.29\times10^{7}$ |
| | 0.814 | $3.21\times10^{7}$ | $9.63\times10^{5}$ | $3.16\times10^{7}$ | $6.47\times10^{7}$ |
| | 0.914 | $2.52\times10^{7}$ | $7.35\times10^{5}$ | $2.48\times10^{7}$ | $5.07\times10^{7}$ |
| $S_1 \rightsquigarrow T_2$ | 0.355 | $1.07\times10^{8}$ | $7.20\times10^{6}$ | $1.07\times10^{8}$ | $2.21\times10^{8}$ |
| | 0.455 | $4.79\times10^{7}$ | $3.45\times10^{6}$ | $4.79\times10^{7}$ | $9.93\times10^{7}$ |
| | 0.555 | $3.17\times10^{7}$ | $2.24\times10^{6}$ | $3.17\times10^{7}$ | $6.56\times10^{7}$ |
| $S_1 \rightsquigarrow T_3$ | -0.053 | $1.68\times10^{5}$ | $3.89\times10^{3}$ | $1.68\times10^{5}$ | $3.40\times10^{5}$ |
| | 0.047 | $9.09\times10^{6}$ | $1.80\times10^{5}$ | $9.15\times10^{6}$ | $1.84\times10^{7}$ |
| | 0.147 | $9.11\times10^{6}$ | $2.14\times10^{5}$ | $9.14\times10^{6}$ | $1.83\times10^{7}$ |

[a] Calculated as the sum of the $k_{ISC}$ for each sublevel.

**Figure S21** Intersystem crossing rate constants of PyrTMEe6, calculated with various singlet-triplet energy differences ΔE(S-T), for each of the $S_1 \rightsquigarrow \{T_1,T_2,T_3\}$ transitions. The bar chart to the right employs a logarithmic scale.

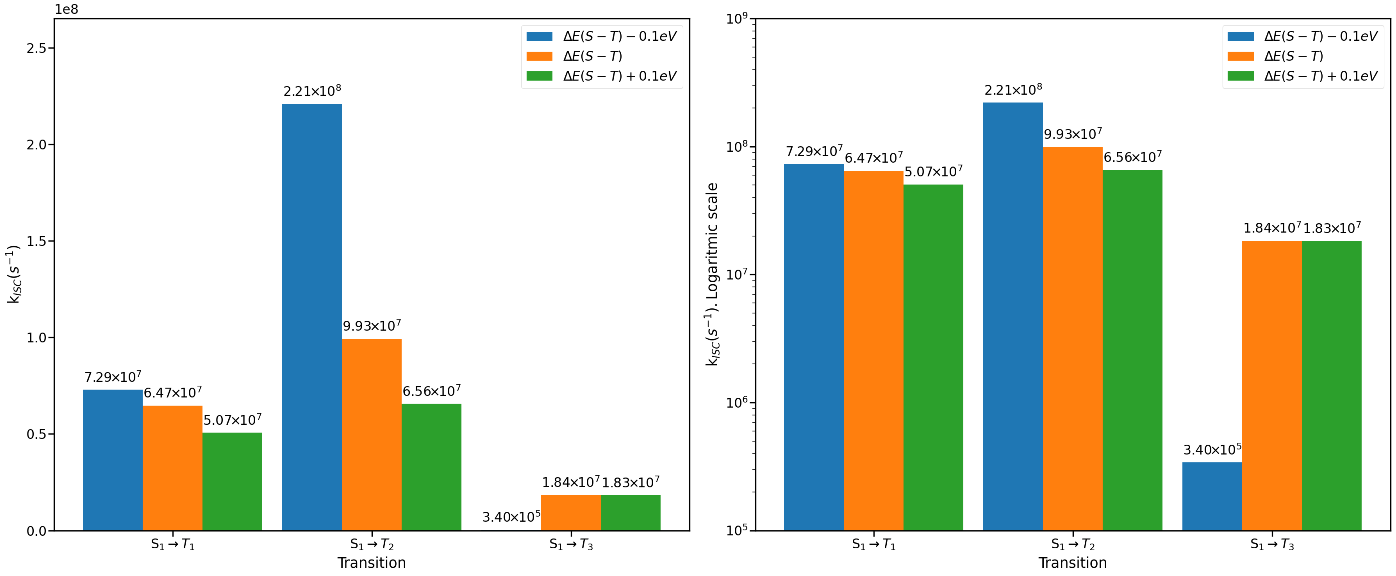


## References


1 Yesica Viviana Blanco Ramírez and Lizeth Tatiana Calderón Hernández. *Síntesis de un derivado de clorofila con potencial aplicación como colorante pancromático en celdas solares*. Bachelor's thesis, Universidad Industrial de Santander, Bucaramanga, Colombia, 2022. Available at https://noesis.uis.edu.co/handle/20.500.14071/12067.

2 Bleidy Muñoz. *Fotooxidación de fenoles utilizando aire y un fotosensibilizador derivado de clorofila*. Bachelor's thesis, Universidad Industrial de Santander, Bucaramanga, Colombia, 2022. Available at https://noesis.uis.edu.co/handle/20.500.14071/11695.

3 Tatiana Tarazona Cáceres. *Unpublished data. Informe avance de proyecto: "Síntesis y caracterización del Hexafluorofosfato de 3'-(1-piridinio) clorina e6 trimetil éster y su complejo metálico con Zn"*. Bachelor's thesis in progress, Universidad Industrial de Santander, Bucaramanga, Colombia, 2024.
